# Unveiling balanced prenatal microbial colonization in amniotic fluid through an integrated culture and sequencing approach

**DOI:** 10.1186/s12967-025-07601-0

**Published:** 2026-01-09

**Authors:** M. González-Rovira, J. A. Sainz-Bueno, L. García-Díaz, C. Martínez-Pancorbo, J. Sánchez, G. Gutiérrez, K. Magoutas, A. Mesías-Pérez, E. Mellado, M. Payne, C. Sousa, M. L. Moreno

**Affiliations:** 1 Department of Microbiology and Parasitology, Faculty of Pharmacy, University of Seville, Seville, Spain; 2 Department of Obstetrics and Gynecology, Faculty of Medicine, University of Seville, Seville, Spain; 3 Institute of Biomedicine of Seville (IBIS), Hospital Universitario Virgen de Valme, CSIC/University of Seville, Seville, Spain; 4 Department of Materno-Fetal Medicine, Genetics, and Reproduction, Institute of Biomedicine of Seville (IBIS), Hospital Universitario Virgen del Rocio, CSIC/University of Seville, Seville, Spain; 5 Department of Surgery, Faculty of Medicine, University of Seville, Seville, Spain; 6 Obstetrics and Gynecology Department, Sagrado Corazón Hospital, Seville, Spain; 7 Department of Genetics, Faculty of Biology, University of Seville, Seville, Spain; 8 Division of Obstetrics and Gynaecology, Medical School, University of Western Australia, Perth, WA Australia

**Keywords:** Prenatal exposome, Amniotic fluid, Intrauterine colonization, Bacterial culture, PacBio 16S rRNA gene sequencing, Illumina shotgun sequencing

## Abstract

**Background:**

The evidence of a low-biomass microbial community in the amniotic fluid (AF) is challenging the traditional concept of a sterile womb. To clarify microbial presence and host responses, a comprehensive, multi-methodological approach is required.

**Methods:**

We designed an optimized culturing strategy that maximized microorganism recovery by implementing differential centrifugation and concentration of AF samples, followed by plating onto four distinct selective media types and incubation under both stringent aerobic (up to two weeks) and prolonged anaerobic (up to four weeks) conditions, including an initial pre-enrichment step in Brain Heart Infusion (BHI) broth for low-abundance organisms. These results were combined with PacBio 16S rRNA gene sequencing, Illumina shotgun metagenomics, and antimicrobial peptides (AMP) detection. Using this approach, we characterized microbial presence in 154 AF samples across gestational stages. Data normality was assessed with the Shapiro-Wilk test, guiding the selection of both parametric and non-parametric tests, and a *p*-value of < 0.05 was considered statistically significant.

**Results:**

We detected culturable microorganisms in 33.1% of samples, with a higher proportion in elective caesarean Sect. (55.0%) compared to amniocentesis (29.5%), suggesting increased microbial load toward term. We applied stringent contamination controls, and repeatedly recovered viable microorganisms *Bacillus*, *Cutibacterium*, *Micrococcus*, and *Staphylococcus*, with *Cutibacterium acnes* and *Staphylococcus epidermidis* common. Both sequencing methods revealed a low-biomass, low-diversity microbial community with high inter-individual variability. Notably, striking microbial discordance in diamniotic twin pregnancies, challenged intrauterine homogeneity. Higher Human Beta Defensin (HBD) -1 levels correlated with absence of culturable bacteria or microbial DNA, while levels of HBD-1, HBD-3, and LL-37 were reduced in *Staphylococcus*-positive samples, suggesting a dynamic interplay between specific bacteria and host defences.

**Conclusions:**

Our findings indicate that viable bacteria and/or DNA can transiently access the prenatal environment microbial balance. We propose a novel perspective of a potential regulatory axis between microorganisms and AMP.

**Supplementary Information:**

The online version contains supplementary material available at 10.1186/s12967-025-07601-0.

## Background

The prenatal environment critically influences the development of the foetal immune system, with lasting implications for immune and metabolic functions, and long-term health outcomes [1, 2]. Foetal immune development plays a fundamental role in preparing the neonates to adapt to microbial environments and establishing lifelong health. Consequently, the timing and nature of prenatal exposure are pivotal in determining their impact on health and development. Although the neonatal innate immune system is capable of immediately responding to pathogens and environmental stimuli at birth, its full maturation requires coordination with the microbiota and other environmental factors [3–6]. Maternal-foetal immune crosstalk involves the transfer of maternal antibodies, ingested allergens, maternal bacteria, microbiota-derived metabolites, immune cells, and cytokines across the placenta and is also facilitated by foetal swallowing [7–10].

The debate regarding uterine sterility during pregnancy and the presence of bacteria in uncomplicated full-term pregnancies persists [11–16]. Nevertheless, the presence of microbes and bacterial DNA in foetal membranes, the placenta, amniotic fluid (AF), and umbilical cord blood prenatally is well-established [17–28]. Both culture-dependent and culture-independent methods have also identified bacterial populations initiating foetal intestinal colonization *in utero* without apparent adverse effects on pregnancy or infant health [29–31].

Although the phenomenon of microbial colonization in the placental foetal compartment is not yet fully understood, several mechanisms have been proposed to explain how microbes may reach this environment. These includes ascending pathways originating from the vagina [32], active sampling of the intestinal lumen by maternal dendritic cells [33], and hematogenous dissemination to the placenta [34]. Furthermore, bacterial translocation via the bloodstream is enhanced during pregnancy and lactation due to weakened mucosal barriers, facilitating bacterial entry into the circulation and placental seeding [35, 36].

Microbial prevalence is often underestimate in cultivation-dependent methods owing to the challenges in culturing anaerobic bacteria or microbes that require specific or unidentified nutrients for growth [15, 37]. Conversely, Next-Generation Sequencing (NGS) technologies face limitations in microbial community profiling due to the difficulty in distinguishing authentic signals from background contamination in low-biomass samples [38–45].

To minimise heterogeneity across studies and improve their comparability to draw more meaningful conclusions, numerous authors have emphasised the need for enhancements in sample collection and processing, as well as the establishment of standardised guidelines for both culture-based and DNA-based sequencing studies [15, 46]. To overcome these limitations and address the need for a comprehensive, multi-methodological approach, our study aimed to optimize and validate culture-based and molecular methods for a robust characterization of the microbial colonization in AF. Specifically, we sought to profile the microbial community in meticulously collected AF samples from two distinct cohorts of pregnant women at different gestational stages from different hospitals, all of whom were free of clinical or analytical suspicion of AF infection and had not initiated labour. Furthermore, we investigated the capacity of AF to support bacterial viability, evaluated the influence of clinical and procedural variables on microbial composition, and assessed the presence of antimicrobial peptides (AMP) and their association with microbial colonization. This approach provides a novel perspective on the foetal microbial composition. We highlight the importance of understanding intrauterine microbial–host interactions for foetal immune development and based on the results of this extensive study, we underscore the significance of a potential prenatal microbial regulatory axis.

## Methods

### Study design and participants

This study involved samples from 148 pregnant women randomly recruited from the Virgen de Valme University Hospital (Seville, Spain), Virgen del Rocío University Hospital (Seville, Spain) and QuirónSalud Hospital (Seville, Spain) to minimize selection bias. Pregnant women scheduled for required procedures (amniocentesis or elective cesarean section) were initially included in a chronological screening list. Following the initial inclusion and exclusion criteria verification, the eligible patients were then thoroughly informed about the study protocol and provided written informed consent prior to sample collection. The maternal age ranged from 18 to 47 years. The exclusion criteria for all pregnant women included the presence of known severe medical conditions, clinical infections, and the use of prescription medications or antibiotics within 2 months prior to study enrolment. Severe medical conditions were defined as those potentially capable of significantly altering the maternal immune status or the foetal environment, including uncontrolled endocrine/metabolic disorders, active systemic autoimmune diseases, severe cardiovascular conditions, chronic infections/immunodeficiency, and severe organ dysfunction.

This study was conducted in accordance with the principles of the Declaration of Helsinki. The study protocol was reviewed and approved by the research ethics committee (REC) (ID/Number: 0452-N-22). Written informed consent was obtained from all participants.

**Fig. 1:**
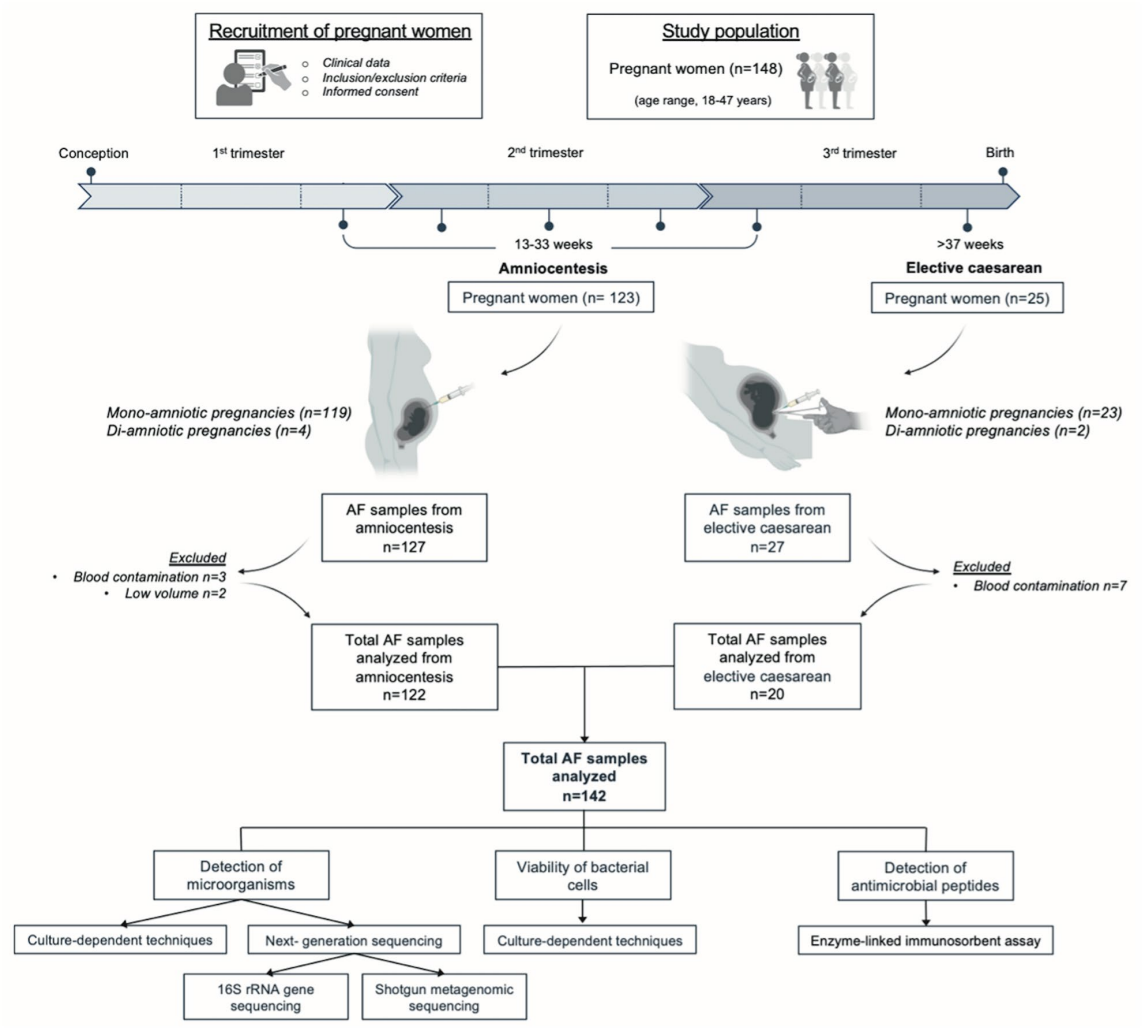
Flowchart of study design, experiments conducted and pregnancy outcomes. AF samples were excluded due to insufficient volume or blood contamination. AF, amniotic fluid

AF samples were collected from women at two different stages of pregnancy: (1) A total of 127 AF samples were collected via amniocentesis between gestational weeks 13 and 33 from 123 pregnant women (including 4 diamniotic pregnancies). The amniocentesis cohort included 56 pregnancies with normal foetuses and 71 pregnancies with foetal anomalies; (2) A total of 27 AF samples were collected at term delivery (> 37 weeks’) from 25 women scheduled for elective caesarean sections (including 2 diamniotic pregnancies) (Fig. 1).

Negative controls were implemented at each stage of the study to address potential contamination. These consisted of extraction process controls using sterile saline solution collected concurrently with amniocentesis or caesarean sections, control of inoculation tools and culture media by inoculating saline solution onto agar plates under the same conditions as the samples, incorporation of sample-free DNA extraction controls in all nucleic acid extractions, and template-free PCR amplification controls.

The inclusion criteria for sample analysis included the absence of blood contamination, and a minimum sample volume of 5 mL. As a result, 12 samples were excluded from the study. Table S1 provides a summary of maternal and foetal characteristics, including maternal age, gestational age at the time of sample collection, and pregnancy data.

### Amniotic fluid sample collection

AF samples (*n* = 154) were collected aseptically by the gynecologist and stored at − 80 °C until analysis, with the assistance of the midwives. The collection of AF samples was conducted as follows: (1) during amniocentesis in the second trimester after abnormal findings on foetal ultrasound or biochemical marker analysis, by inserting a needle through the abdomen into the uterus to remove a 5–10 mL AF sample and, (2) during elective (planned, with no ongoing labor) caesarean delivery, at the time of caesarean section by aspirating through intact amniotic membranes using a sterile probe and 20-mL syringe.

To mitigate the risk of contamination, strict protocols were implemented throughout the collection and transport process. These protocols were based on aseptic techniques, use of sterile single-use equipment, and immediate refrigeration of samples to minimise environmental exposure. The personnel followed standardised procedures, including wearing protective equipment and operating in a controlled environment. This meticulous approach ensured the integrity of the AF samples and preserved their suitability for subsequent microbial analyses.

### Optimization of procedures for microbial culture analysis of AF samples

#### Concentration of AF samples

Each AF sample was divided into 2.5 mL aliquots and concentrated 2-, 3-, 4-, or 5-fold by centrifugation (Heraeus Biofuge Pico, Thermo Scientific, Waltham, MA, USA). To optimise the centrifugation parameters without compromising bacterial viability, AF concentrates were centrifuged at 1,250, 3,850, 9,500, and 13,250 × g, for durations ranging from 0 to 15 min at room temperature. After centrifugation, the pellets were resuspended in their respective supernatants and then they were discarded, confirming the absence of significant bacterial material by the lack of growth after culture.

#### Culture media and incubation conditions

Pellets of each AF concentrate sample (100 µL) were inoculated and streaked in duplicate onto four media types: Brain Heart Infusion (BHI) agar (Millipore, Merck, Darmstadt, Germany), Columbia Blood (CB) agar (VWR Chemicals, Barcelona), Gifu Anaerobic Medium (GAM) agar (Nissui Pharmaceutical, Japan), and MacConkey (MC) agar (Millipore, Merck, Darmstadt, Germany). Culture media were selected based on previous studies used to isolate microorganisms from the AF samples [18, 47–49]. Plates were incubated at 37 °C under aerobic conditions (BHI, CB, and MC) and anaerobic conditions (BHI, CB, GAM, and MC), using AnaeroGen 2.5 L gas-generating sachets (Thermo Scientific, Oxoid), which create an anaerobic atmosphere (< 1% O_2_, ~ 10–15% CO_2_) inside an Oxoid jar. The plates were inspected daily for bacterial growth for up to two weeks under aerobic conditions and for four weeks under anaerobic conditions. To maintain stringent anaerobic conditions throughout the incubation period, the anaerobic environment was continuously monitored, and gas-generating sachets were replaced weekly.

#### Pre-enrichment step

To recover low-abundance or fastidious organisms, a 200 µL aliquot of each 5-fold concentrate was inoculated into 4 mL sterile BHI broth and incubated aerobically at 37 °C for 48 h with gentle agitation (200 rpm). After enrichment, 100 µL was sub-cultured onto BHI, CB, GAM, and MC plates as described above.

### Bacterial culture and 16S rRNA gene sequencing

For bacterial culture from the AF samples, BHI, CB, GAM, and MC agar were used. The samples were plated and incubated at 37 °C under either aerobic or anaerobic conditions to maximize the detection of cultivable microorganisms. Each strain was subcultured for an additional 48 h under the same conditions, and DNA was extracted using the GeneJET Genomic DNA Purification Kit (Thermo Scientific). Subsequently, PCR was performed on each isolated bacterial colony using universal bacterial primers targeting the 16S ribosomal RNA (rRNA) gene (27 F/1492R). Negative control samples included PCR amplification reagents and DNA-free water that were subjected to the extraction protocol. Gel electrophoresis confirmed the presence of amplicons of the expected size (~ 1500 bp). The PCR products were purified and sequenced by STAB VIDA (Costa da Caparica, Portugal) using pairwise Sanger sequencing. Sequence chromatograms were inspected manually using ChromasPro 2.2 to ensure the accuracy of base calls, and the resultant sequence was entered into the National Center for Biotechnology Information (NCBI) Basic Local Alignment Search Tool (BLAST) to determine bacterial taxonomy. Sequences with > 99% similarity and > 99% query coverage were considered to belong to the same species.

### Assessment of the capacity of AF samples for bacterial growth

We designed a series of experiments using AF samples confirmed to lack detectable cultivable microorganisms, aiming to test the intrinsic capacity of AF to support bacterial viability and proliferation. By employing forced, non-physiological conditions atypical of the in utero environment, we sought to detect dormant or very low-abundance microbes that typically remain undetected under standard in utero or conventional culture conditions.

AF samples were individually inoculated with four bacterial species (*B. subtilis*, *C. acnes*,* M. luteus* and *S. epidermidis*) in sterile microcentrifuge tubes and incubated at 37 °C on a shaker at 200 rpm under both, aerobic and anaerobic conditions, to ensure uniform bacterial growth. Each microorganism was cultured in liquid BHI medium until it reached an optical density (OD) of 0.5, corresponding to the exponential growth phase, and a 1:10 dilution of the culture was used to inoculate the AF samples. To assess bacterial growth, the absorbance at 600 nm was measured at time 0 and after different incubation times (6, 12, 24, 48, 72, and 96 h) using Nunc cell culture plates (polystyrene 96-well plates, Thermo Scientific). Negative controls containing only water and culture medium without sample were included and subjected to the same procedure as the inoculated samples. To quantify the colony-forming units (CFU) of the bacterial species that survived and proliferated in the AF after inoculation, these samples were cultured in BHI medium at 37 °C under both aerobic and anaerobic conditions.

### Nucleic acid extraction and sequencing

DNA was extracted from each AF sample using two different protocols in parallel, with the aim of selecting the kit with the greatest yields and lowest number of contaminants: (1) QIAamp PowerFecal Pro (PFP) DNA kit (QIAGEN Pty Ltd., Hilden, Germany) and (2) QIAGEN MagAttract Microbial (MA) DNA kit (QIAGEN Pty Ltd) on the Kingfisher DUO platform (Thermo Scientific) following the manufacturer’s instructions. A blank extraction control, consisting of DNA-free water was used for each extraction batch. The integrity and purity of the extracted nucleic acids were assessed via NanoDrop ND-1000 spectrophotometer and agarose gel electrophoresis. DNA concentrations were quantified with a Qubit 4.0 Fluorometer (Thermo Scientific).

**Fig. 2:**
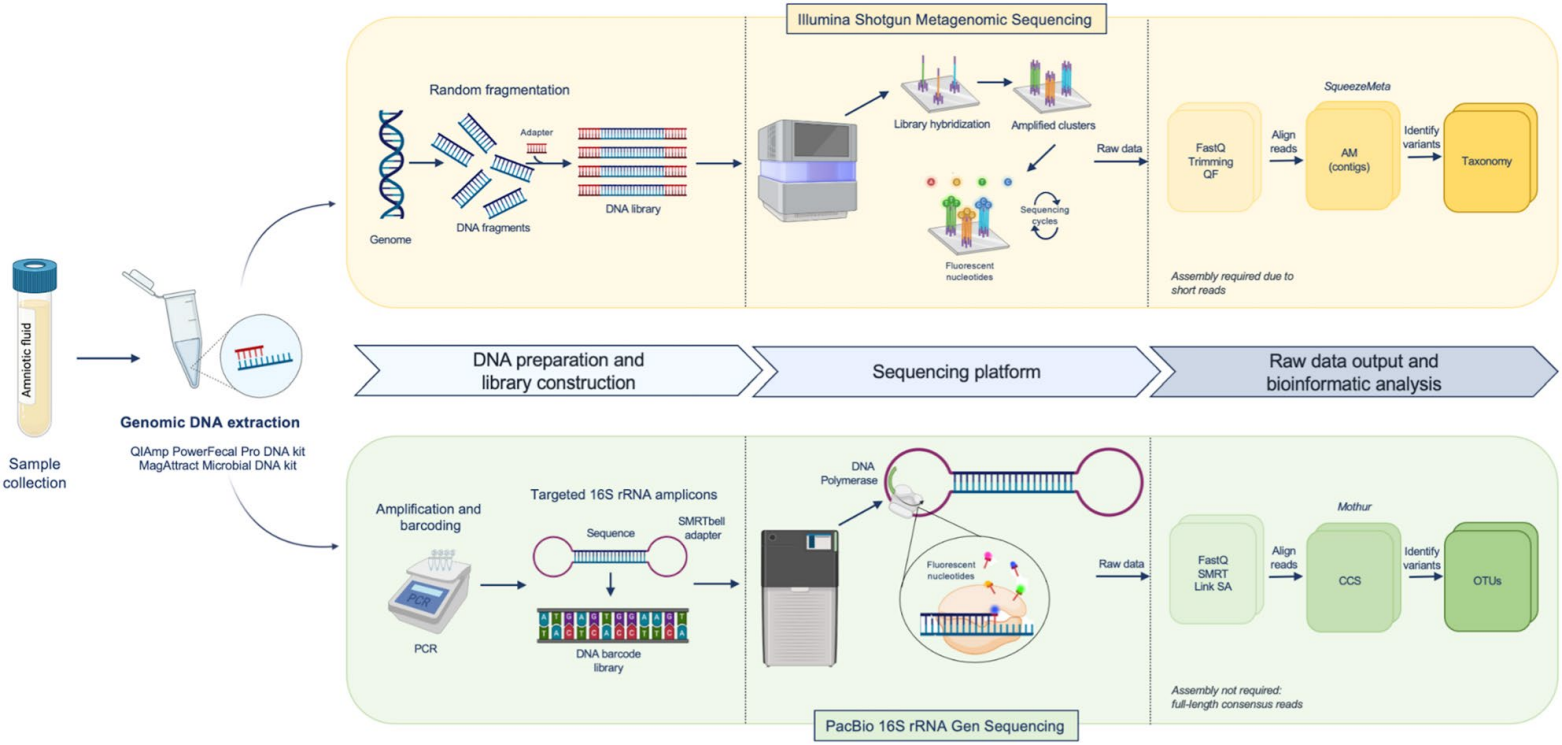
Workflow of NGS techniques for DNA analysis in AF using two metagenomic approaches. It compares whole-genome shotgun sequencing on the Illumina platform and targeted 16S rRNA gene sequencing on PacBio. AF, amniotic fluid; AM, assembled metagenome; CCS, circular consensus sequencing; Next-Generation Seguencing, NGS; OTUs, operational taxonomic units; QF, quality filtering; SA, sequencing analysis

DNA was analysed using two NGS techniques (Fig. 2). First, shotgun metagenomic sequencing was performed using a NovaSeq 6000 system (Illumina, USA) by Novogene Europe (Cambridge, UK). Library preparation and sequencing generated 150 bp paired-end reads. Raw reads were processed using the NovogeneAIT Genomics pipeline to remove adapters, low-quality sequences, and ambiguous bases. The cleaned reads were subsequently used for bioinformatics analysis. Second, a 16S rRNA gene amplicon sequencing strategy was utilised, with amplicons obtained by PCR using asymmetric combinations of 5 forward primers and 24 reverse primers with barcodes (27 F/1492R primer set), as previously described by Stinson et al. (2019a) [23]. PCR reactions were prepared using dsDNase-treated mastermixes (ArcticZymes) to minimise DNA contamination. The PCR conditions included 35 cycles of amplification. Amplicons were visualised using a High-Resolution Kit on a QIAXcel capillary gel electrophoresis system and pooled on equimolar concentrations based on band quantification within the target size range (1336–1743 bp). Subsequently, pooled samples were concentrated using NucleoMag NGS Clean-up and Size Select magnetic beads (Macherey Nagel) according to the manufacturer’s instructions and eluted in 75 µL volumes. The purified DNA (500 ng) of was used for library preparation at the Australian Genome Research Facility (AGRF) (Melbourne, Australia). SMRTbell adapters were ligated to the barcoded PCR products, and the library was sequenced using the PacBio Revio system on a single SMRT cell.

### Detection of HBD-1, HBD-2, HBD-3, HNPs 1–3, and LL-37 by ELISA

A specific and sensitive enzyme-linked immunosorbent assay (ELISA) was used to detect the AMP in the AF samples, including α-defensins (Human Neutrophil Peptides, HNPs 1–3), β-defensins (Human Beta Defensin, HBD 1–3), and cathelicidins (LL-37). HBD-1, HBD-2 and HBD-3 kits from Aviscera Bioscience (Cat#SK00858–06, Santa Clara, CA, USA). HNPs1-3 and LL-37 were detected using Human ELISA Kits from Hycult Biotech (Uden, Netherlands). All ELISA kits have been validated for use in prior studies [50–52]. AMP concentrations were calculated by interpolation from individual standard curves composed of purified defensins (0.97 to 125 pg/mL for HBD-1, 31.25 to 2,000 pg/mL for HBD-2, 39.5 to 2,500 pg/mL for HBD-3, 156 to 10,000 pg/mL for HNPs 1–3, and 0.1 to 156.25 ng/mL for LL-37). The sensitivity of the assay was 0.3 pg/mL for HBD-1, 7.0 pg/mL for HBD-2, 10.0 pg/mL for HBD-3, 19.46 pg/mL for HNPs 1–3, and 0.14 ng/mL for LL-37.

### Data analysis

#### Bacterial isolate 16S rRNA gene sequencing

The 16S rRNA gene sequences obtained from the isolated species were assembled and edited using ChromasPro 2.2. Phylogenetic and molecular evolutionary analyses were conducted using MEGA X [53, 54]. Multiple sequence alignment was performed with ClustalW [55]. Phylogenetic trees were constructed using the Neighbor-Joining (NJ) algorithm [56]. The bootstrap test was applied with 1000 replicates to estimate the reliability of the tree topology, and the percentage of replicate trees in which the associated taxa clustered together is shown next to the branches. Evolutionary distances were computed using the Kimura 2-parameter model [57], expressed as the number of base substitutions per site. All ambiguous positions were removed for each sequence pair using the pairwise deletion option.

#### Shotgun metagenomic sequencing

The cleaned metagenomic reads generated by shotgun sequencing on a NovaSeq 6000 platform (Illumina, USA) were analysed using the SqueezeMeta pipeline [58]. The reads were assembled into contigs using the MEGAHIT assembler [59] optimised for complex metagenomic datasets. Clean reads were then mapped back to the assembled contigs using Bowtie2 [60] to estimate the coverage and relative abundances. Contigs were grouped into genomic bins based on their sequence composition and coverage profiles, using a consensus approach combining MetaBAT [61] and COmbat CO-Normalization Using controls (COCONUT) [62]. The taxonomic classification of contigs and bins was performed within SqueezeMeta using the DIAMOND aligner [63] against the GenBank nr database. Functional annotation was carried out by mapping predicted open reading frames (ORFs) to the KEGG database [64] to infer potential metabolic pathways and gene functions. All downstream analyses and data visualization were performed using the SQMtools R package [65], enabling seamless exploration of the taxonomic and functional structure of microbial communities from SqueezeMeta outputs. For metabolic reconstruction and genomic validation of *Phyllobacterium*, the contigs assigned to this genus were analysed with the MiGA online platform to assess genome quality and taxonomic affiliation [66]. The contigs were then annotated with the RAST server for functional gene prediction and subsystem-based annotation [67].

#### PacBio full-length 16S rRNA gene sequencing

PacBio raw sequence reads were processed using the PacBio SMRT Link analysis software v6.0 to generate circular consensus sequences (CCS) with a minimum of three full passes and an accuracy threshold of 99.5%. The sequences were then demultiplexed according to the asymmetric barcoding strategy. Sequence data processing was performed using the Mothur software package (version 1.48.0) [68]. Reads were filtered by length, ranging from 1336 to 1743 bp, and further processed to exclude sequences containing homopolymers longer than nine bases. Alignment was performed against the SILVA reference alignment database (v138) [69]. Chimeric sequences and those mapping to non-bacterial taxa were subsequently removed. Sequences were clustered into OTUs by calculating pairwise distances, followed by clustering with a similarity cut-off of 0.03 using the cluster.split command. Alpha diversity was assessed using richness (number of different OTUs) and Shannon diversity. Beta diversity was evaluated using PERMANOVA with Bray-Curtis distances implemented within the vegan package of R. For the differential abundance analysis, a prevalence threshold of 10% with a minimum count of three reads was applied. Taxonomic assignment of the OTUs of interest was performed using BLAST [70, 71], with sequence identity scores of > 99% considered reliable matches at both the genus and species levels.

Data processing and statistical analyses were performed using Microsoft Excel, SPSS software version 19 (SPSS Inc., IBM Corporation, Armonk, NY, USA), and R Studio (v. 2024.09.1 + 394) (R Core Team R, 2020). Data normality was assessed with the Shapiro-Wilk test. Non-parametric tests were applied when necessary, and corrections for multiple comparisons were performed. An unpaired two-tailed Student’s t-test was used to assess statistical significance, with a p-value of < 0.05 considered significant. The Mann-Whitney U test was employed to compare quantitative variables in independent groups, while Wilcoxon signed-rank tests were applied for comparisons within dependent groups.

## Results

### Optimisation and validation of culture methodologies for amniotic fluid microbial recovery

In this study, we improved and fine-tuned laboratory procedures to maximize the growth and isolation of microorganisms from AF samples using a subset of random AF samples (*n* = 10) to cover different clinical and technical conditions by colony counting on plates, through colony counting on plates and with a rigorous protocol to minimize contamination (see Methods).

Of the various concentration and centrifugation conditions tested, a 5-fold concentration of AF combined with centrifugation at 9,500 × g for 12 min exclusively yielded colony growth.

Four media types were tested using AF samples under both aerobic and anaerobic conditions. Colonies were observed in grew on all media except MC agar, which was subsequently excluded. Most aerobic colonies developed within 48–96 h, although some required up to 5 days. Pre-enrichment in BHI medium was proved crucial for the detection of slow-growing or otherwise undetectable microorganisms. No microbial growth was observed on the plates from any of the negative control samples used in this study.

### Profiling of culturable bacteria in amniotic fluid samples: a comprehensive analysis across gestational stages

Following the successful optimization of culture methods, microorganisms were investigated and successfully detected in AF samples (*n* = 142) collected at different gestational stages of pregnancy. Twelve AF samples were excluded from the analysis due to blood contamination or insufficient volume (Fig. 1).

**Fig. 3:**
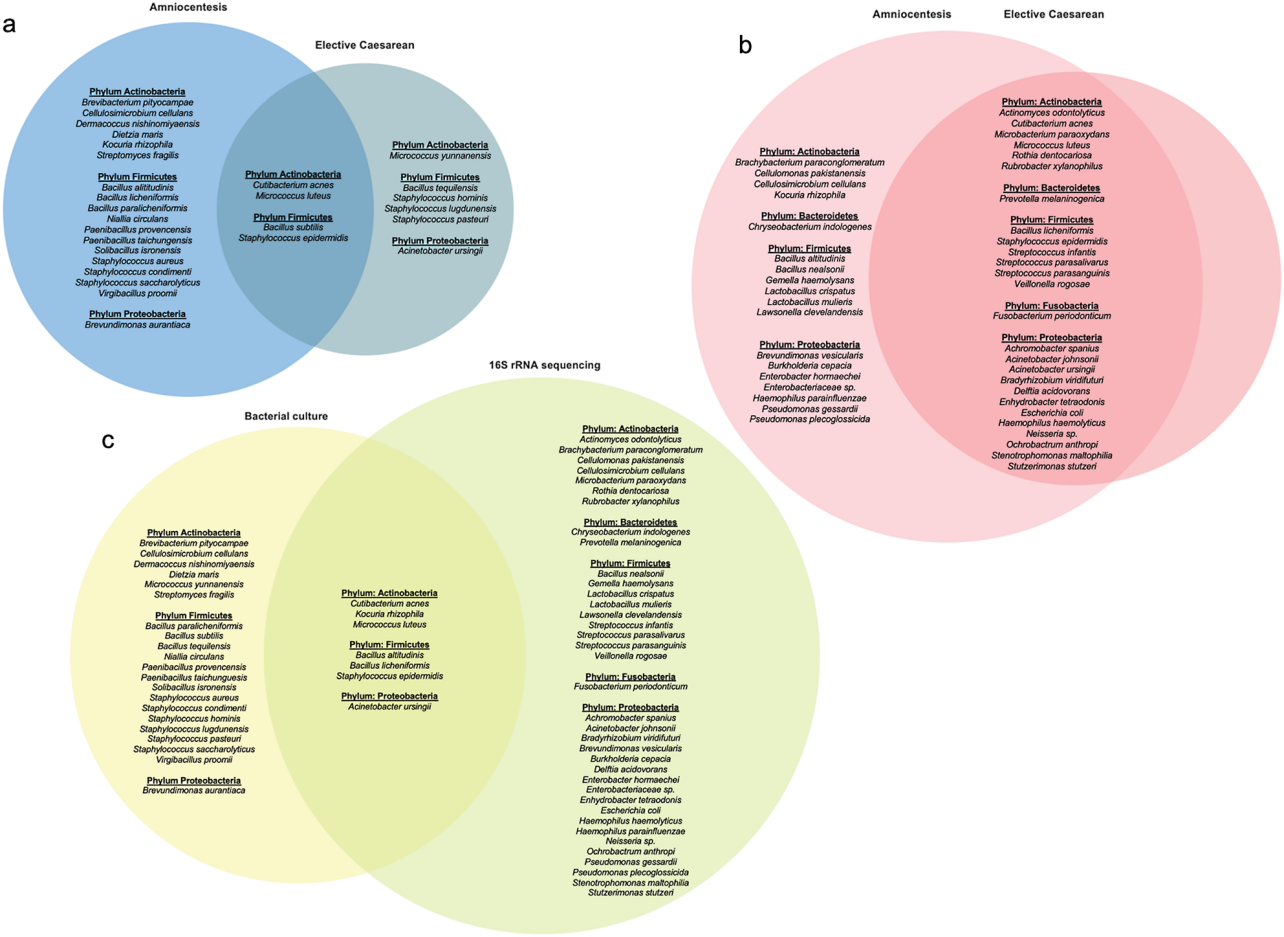
Venn diagram illustrating overlap between cultivable and 16S rRNA-identified bacterial species in AF samples. **a** Cultivable bacterial species isolated from AF samples collected via amniocentesis and elective caesarean sections. **b** Bacterial species identified by 16S rRNA sequencing from AF samples collected via amniocentesis and elective caesarean sections. **c** Intersection of cultivable bacterial species and those identified by 16S rRNA sequencing in AF samples from both procedures. AF, amniotic fluid

Of the 142 AF samples analysed, 33.1% contained culturable microorganisms. Specifically, 29.5% (36/122) of amniocentesis samples and 55.0% (11/20) of caesarean section samples tested positive for the presence of culturable microorganisms. To identify bacteria recovered from the AF samples using culture from both gestational periods, the 16S rRNA gene was amplified. Species in the Venn diagram (Fig. 3A) representing the closest identified bacterial matches (≥ 99% homology). Microorganisms belonging to the phyla Actinobacteria, Firmicutes and Proteobacteria were identified. Notably, species such as *Bacillus subtilis*, *Cutibacterium acnes*, *Micrococcus luteus*, and *Staphylococcus epidermidis* were present in the AF samples from both gestational periods. Regarding abundance, *C. acnes*, *M. luteus*, and *S. epidermidis* were predominant in the second trimester samples, whereas *Staphylococcus lugdunensis*, *B. subtilis*, and *S. epidermidis* were most prevalent in term deliveries.

Given that previous studies have suggested the potential for microbiological contamination during AF samples analysis, we compared the 16S rRNA gene similarity of individual isolates and included negative controls processed in parallel to assess that possible contamination. A thorough phylogenetic analysis (Fig. S1) was conducted on the 53 isolates obtained from AF samples collected through both amniocentesis and caesarean sections, categorised by the hospital where each sample was collected. With the exception of *B. subtilis*, all bacterial species were isolated from women across all three participating hospitals. Phylogenetic analysis revealed several patterns within these species. Isolates from caesarean sections were often clustered with those from amniocentesis, indicating no distinct grouping based on gestational age or hospital of origin. Across all species studied, isolates formed clear, distinct clusters and branches, with no correlation found related to the sample source or collection site. Negative controls showed no microbial growth, supporting the validity of the isolates obtained.

Next, we assessed microbial colonization in AF samples by counting the isolated colonies (Fig. S2). Of the culture-positive amniocentesis plates, 96.7% yielded a single colony suggesting limited microbial colonization or low diversity consistent with the expected low microbial load of AF during mid-pregnancy. In contrast, caesarean section samples showed more varied patterns: five samples had a single colony, whereas 54.54% exhibited multiple colonies, ranging from 2 to 23 per plate. In total, 147 microbial colonies were identified in the AF samples: 82 from amniocentesis (47 aerobic and 35 anaerobic) and 65 from caesarean Sect. (12 aerobic, and 53 anaerobic). Colony counts differed significantly between aerobic and anaerobic conditions (*p* = 0.0005), with caesarean section samples showing a higher number of colonies under anaerobic conditions. This significant difference indicates a predominance of anaerobic bacteria in caesarean AF samples. No significant differences in colony counts were observed between BHI and CB media.

The absolute abundance of culturable bacterial species per AF sample was also analysed to provide an overview of the microbial community diversity in AF samples obtained through amniocentesis and elective caesarean section. A single species was isolated from 47.22% (17/36) of the culture-positive amniocentesis samples. Another 30.56% (11/36) contained two species, 8.33% (3/36) contained three species, and 13.89% contained more than three species. In contrast, among the caesarean section samples, 45.45% (5/11) contained a single species, 36.36% (4/11) contained two species and 18.18% (2/11) had more than three species. The highest microbial diversity was observed in one caesarean sample, where six species were identified, showed the highest number of colonies for each of these bacterial species.

### Evaluating the capacity of amniotic fluid to support bacterial viability and proliferation under controlled experimental conditions

The intrinsic capacity of AF to support bacterial viability was assessed using seven AF samples (AF_18, AF_36, AF_37, AF_41, AF_42, AF_43 and AF_44) previously verified to be free of detectable culturable microorganisms. The sterility of the experimental procedures was confirmed as all parallel negative controls (water and culture medium) consistently yielded no growth.

**Fig. 4:**
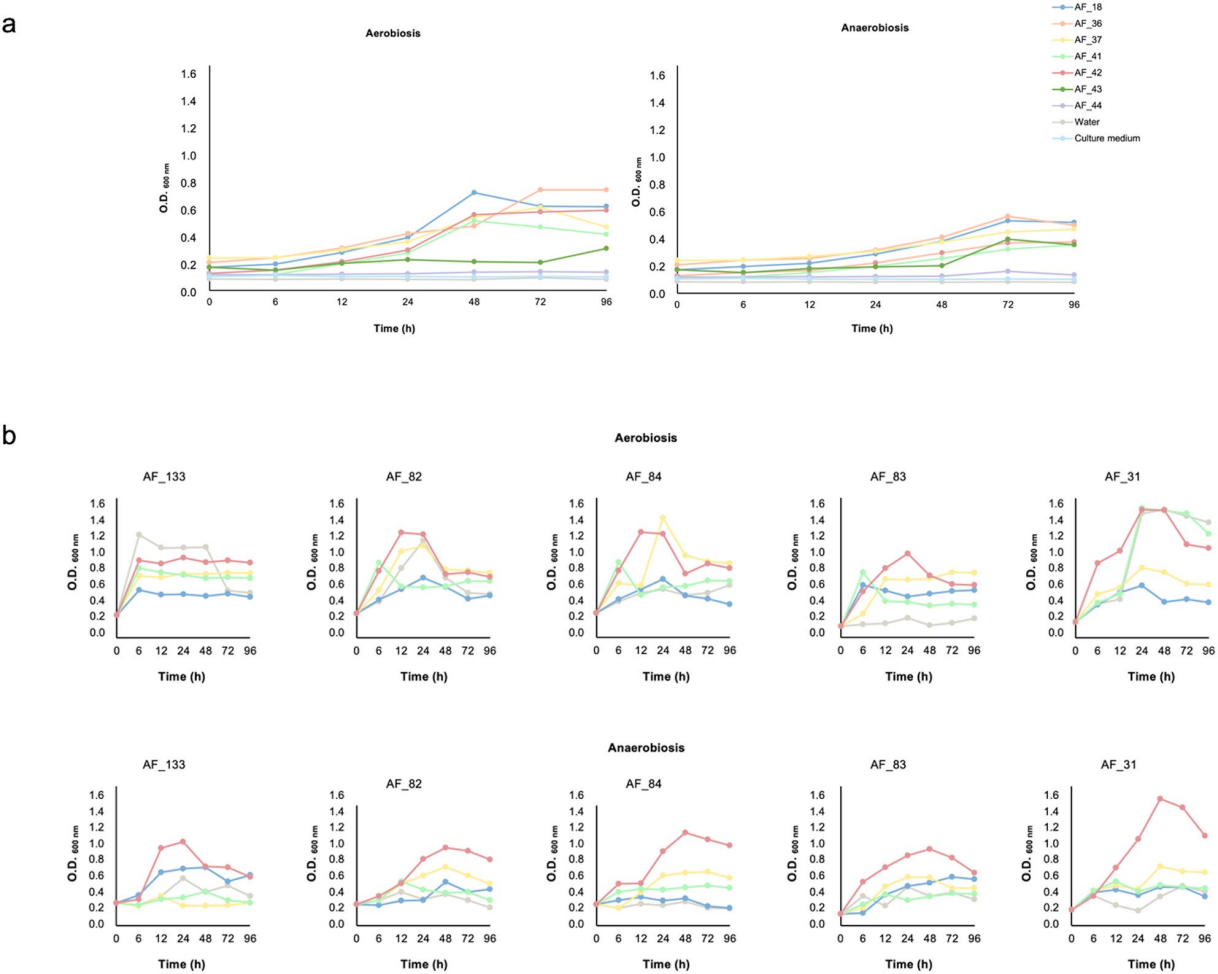
Bacterial growth dynamics in AF samples. **a** Optical density (O.D.600 nm) profiles of AF samples incubated without bacterial inoculum under aerobic and anaerobic conditions. **b** Growth curves of *Bacillus subtilis* (yellow), *Cutibacterium acnes* (red), *Staphylococcus epidermidis* (blue), and *Micrococcus luteus* (green) cultured in AF samples and AF samples without bacterial inoculum (grey), showing bacterial proliferation over time under both aerobic and anaerobic conditions. AF, amniotic fluid

An increase in absorbance was observed in these AF samples after 96 h, suggesting the presence of microorganisms at low or dormant levels, which were able to proliferate to detectable concentrations under the applied incubation and the experimental conditions (Fig. 4A). Subsequent plating of the enriched AF sample on solid media led to the isolation of *Amycolatopsis pretoriensis* and *Cellulosimicrobium funkei*, underscoring the value of this approach for discovering low-abundance or slow-growing microbes in AF.

In a subsequent experiment, we used five AF samples (AF_31, AF_82, AF_83, AF_84 and AF_133) in which no culturable bacteria were detected under the initial conditions, to assess their ability to support the growth of bacterial isolates. For this, four of the most isolated species from the previous solid medium culture assay (*B. subtilis*, *C. acnes*, *M. luteus*, and *S. epidermidis*) were individually inoculated into AF samples using sterile microcentrifuge tubes. Negative water controls were again included and remained sterile throughout the experiment. Our results indicated substantial variations in microbial growth among the tested species, contingent on the specific AF sample serving as the culture medium (Fig. 4B). Furthermore, differential growth patterns were evident among the various bacterial species within the same AF sample. Of the four species analysed, *C. acnes* consistently demonstrated the highest growth, whereas *S. epidermidis* displayed the lowest proliferative capacity across all AF samples.

### Metagenomic profiling of the amniotic fluid via full-length 16S rRNA gene sequencing

Metagenomic studies on AF critically require rigorous controls due to the challenges of low microbial biomass and persistent contamination risk, particularly from reagents (“kitome”).

In this study, we first assessed the influence of two DNA extraction kits on bacterial DNA detection in 18 randomly selected AF samples, evaluating levels of contaminants, and distinguishing true microbial signals from background noise using negative controls.

Full-length 16S rRNA gene sequencing was performed to evaluate the performance of each kit in terms of Operational Taxonomic Unit (OTU) recovery and the extent of kitome contamination [39, 40, 72]. The inclusion of blank extraction controls in each batch confirmed the success of the comparative approach (Table 1).

**Table 1:**
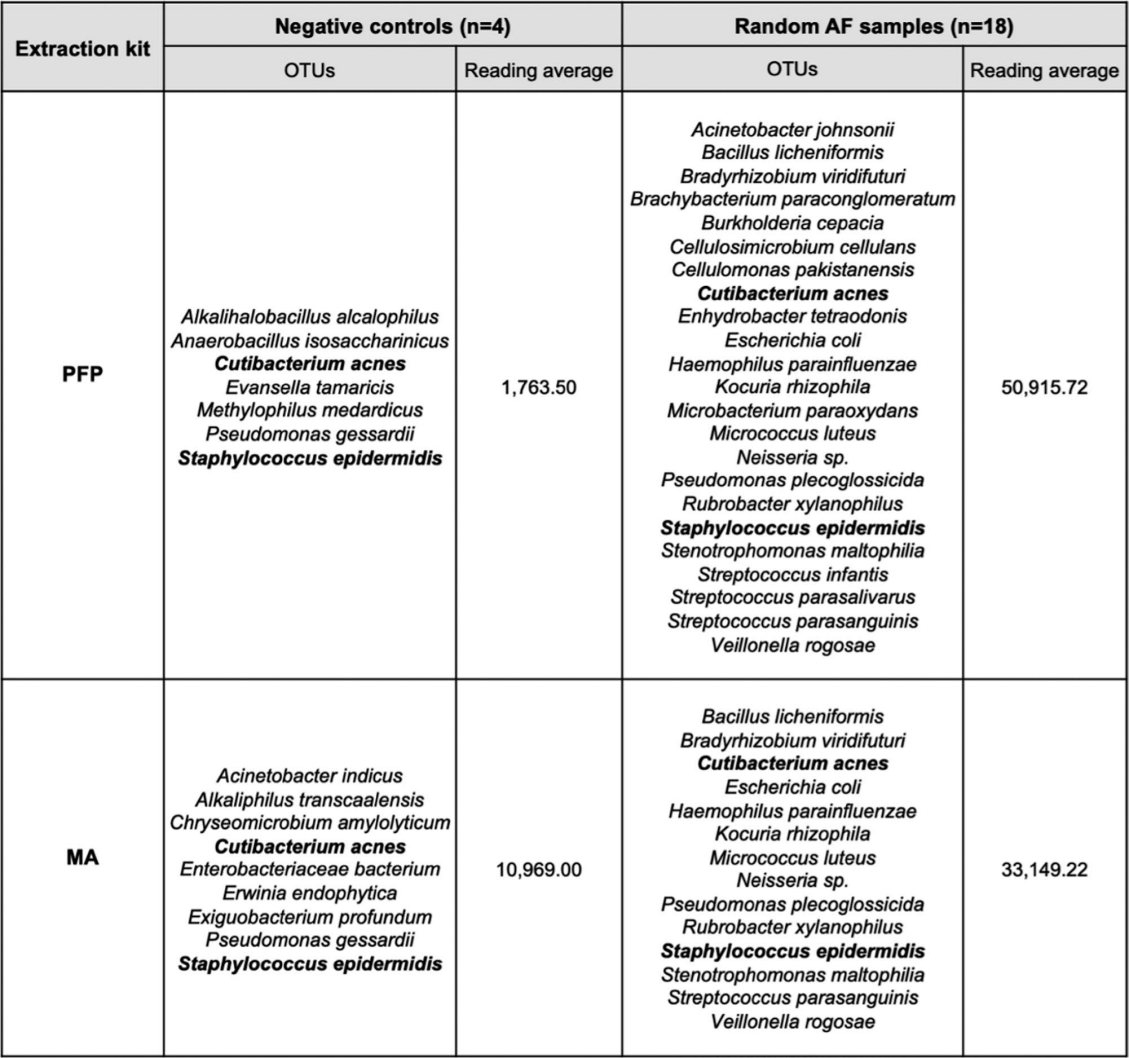
Comparison of bacterial taxa in negative controls and random AF samples using two kits

PFP and MA DNA extraction kits, were applied to assess potential variability in microbial profiles identified by 16S rRNA sequencing. Species in bold correspond to those detected in both, negative controls and random AF samples. AF, amniotic fluid; MA; Mag Attract; OTUs, Operational Taxonomic Units; PFP, PowerFecal Pro

All negative controls quantified fell below the detection limit, indicating minimal contamination during DNA extraction. As shown in Table 1, the PFP kit produced a lower average read count in negative controls compared to the MA kit, suggesting reduced background contamination. Reads corresponding to several bacterial taxa were detected exclusively in the AF samples and not in the negative controls. The only two species shared between the AF samples and negative controls were *C. acnes* and *S. epidermidis*. Notably, the PFP kit yielded a higher average read depth in AF samples, indicating improved sensitivity for detecting microbial DNA. To evaluate the potential contamination introduced during PCR amplification, four negative PCR controls were included, which yielded an average of 140.75 reads. Only reads corresponding to three bacterial species were detected in these controls: *C. acnes*, *Exiguobacterium profundum*, and *S. epidermidis*, although not a significant level in relation to the overall reads obtained; among these, only *C. acnes* and *S. epidermidis* were also identified in AF samples (Table 2).

**Table 2:**
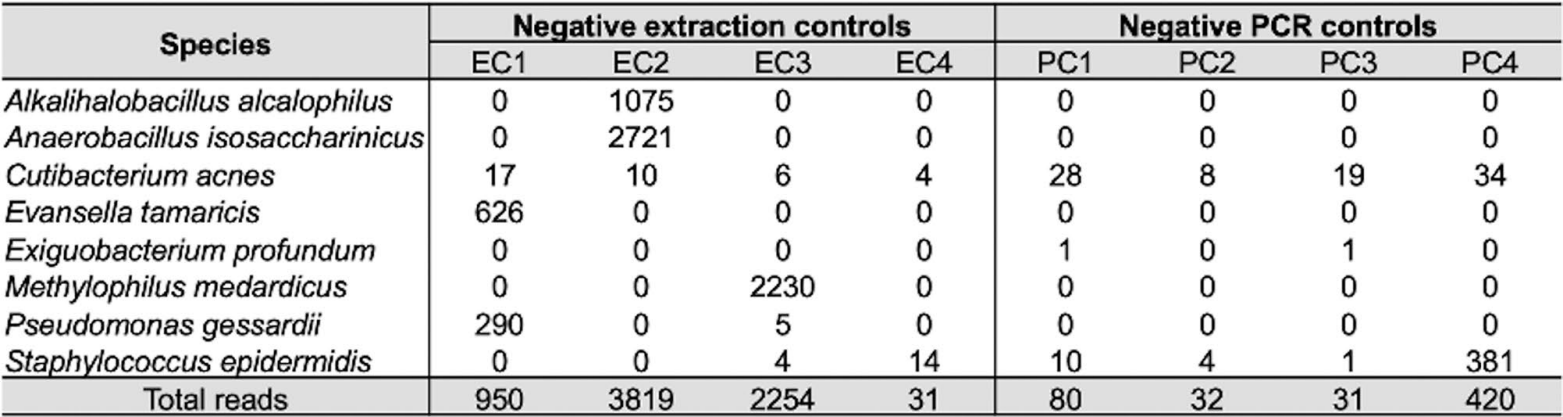
16S rRNA reads from negative extraction and PCR controls

Sequence reads were obtained from negative extraction controls (EC1–4) with the PFP kit, and from negative PCR controls (PC1–4). EC, extraction controls; PC, PCR controls; PFP, PowerFecal Pro

Subsequently, 73 AF samples were sequenced using the PFP kit. To standardize sequencing depth, subsampling was performed to 1,413 reads, based on the smallest library size, excluding negative controls. We obtained an average of 53,043.82 reads per AF sample collected via amniocentesis, corresponding to an average of 5.86 OTUs per sample. Similarly, AF samples collected during elective caesarean sections yielded an average of 34,665.15 reads and 4.38 OTUs per sample. Overall, the AF samples exhibited a bacterial community characterised by low abundance and limited diversity. Notably, all bacterial species identified in the AF samples collected during the caesarean sections were also present in those obtained by amniocentesis (Fig. 3B). The sequenced bacterial phyla included Actinobacteria, Bacteroidetes, Firmicutes, Proteobacteria, and Fusobacteria.

To assess the consistency and complementarity between traditional culture-based methods and culture-independent 16S rRNA gene sequencing, we compared the bacterial taxa identified by each approach. As shown in Figs. 3C and 65 bacterial taxa were detected across both methods. Of these, seven taxa were identified by both culture and 16S rRNA gene sequencing, and 37 taxa were detected exclusively by sequencing. The taxa shared between both methodologies included representatives of the phyla Actinobacteria (*Kocuria rhizophila*, *C. acnes* and *M. luteus*), Firmicutes (*Bacillus licheniformis*, *Bacillus altitudinis* and *S. epidermidis*), and Proteobacteria (*Acinetobacter ursingii*). Culture-based methods identified several unique taxa, primarily within the phyla Firmicutes and Actinobacteria. Conversely, 16S rRNA gene sequencing offered broader phylogenetic coverage, detecting additional phyla such as Bacteroidetes (*Chryseobacterium indologenes* and *Prevotella melaninogenica*) and Fusobacteria (*Fusobacterium periodonticum*), previously not identified by the culture. Taken together, the direct comparison between both methodologies showed that, although taxonomic overlap was limited, integrating culture and sequencing datasets expanded the overall taxonomic spectrum. This integration encompassed both viable bacteria and those detected exclusively at the sequence level, thereby underscoring the complementary value of parallel approaches for microbial characterization.

**Fig. 5:**
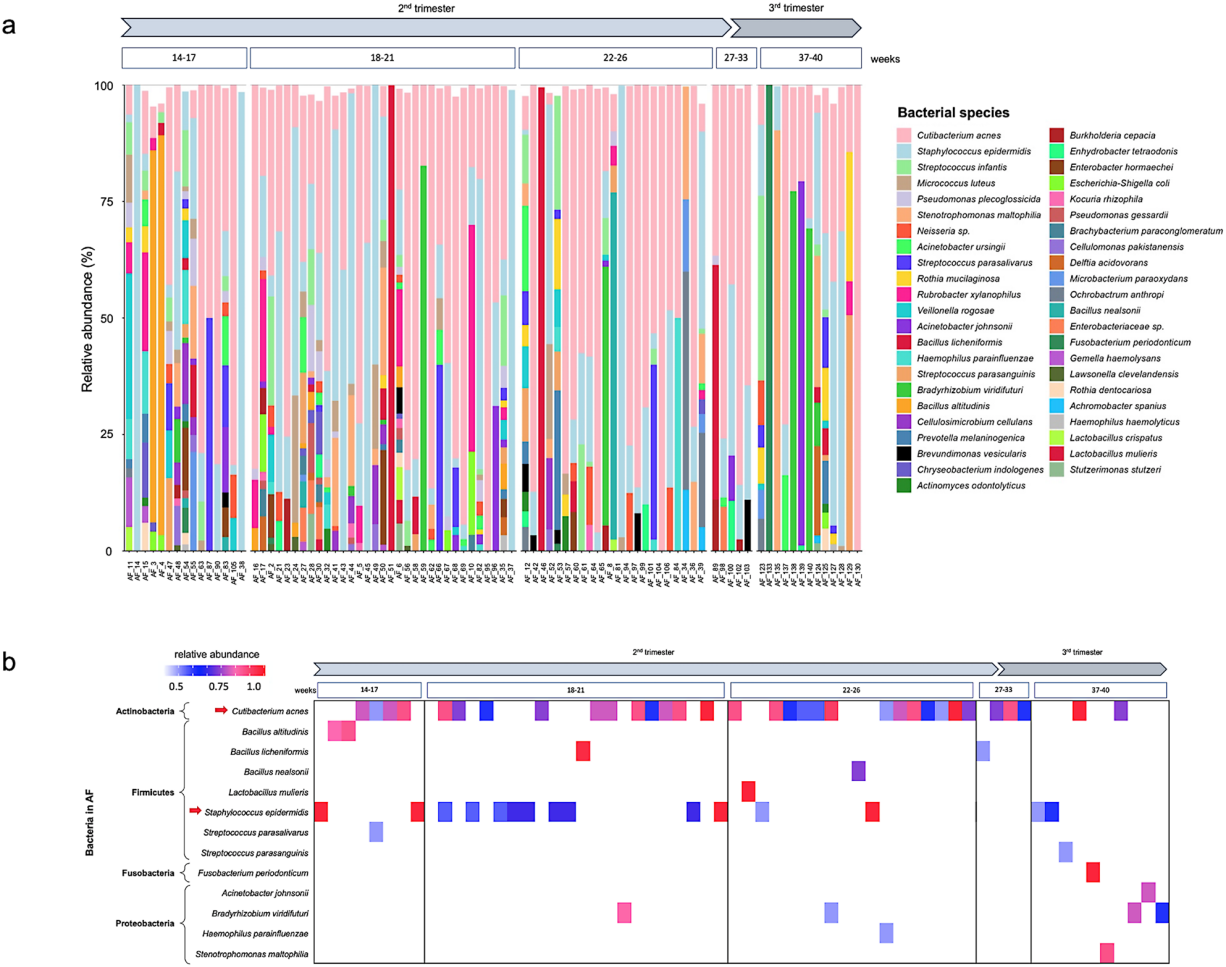
Relative abundance of bacterial species detected in AF samples by 16S rRNA gene sequencing. **a** Percentage of bacterial species categorised by gestational age and weeks of pregnancy. **b** Heatmap of species with > 0.5 relative abundance. High bacterial relative abundance is represented with color gradation which increases toward red, while low bacterial relative abundance tends toward blue; the arrow indicates the most abundant species identified. AF, amniotic fluid

The relative abundance of bacterial species detected in AF samples across different gestational age groups was also analysed (Fig. 5A). The microbial profiles exhibited considerable inter-individual variability, with no single taxon predominating consistently across all samples. *C. acnes* and *S. epidermidis* were among the most consistently abundant species across the second and third trimesters, corroborating their frequent detection by both culture and sequencing methods. *C. acnes* was present in 84.27% of the samples, while *S. epidermidis* was detected in 76.40% of the samples, highlighting their widespread occurrence within the cohort.

To further investigate the composition of the dominant microbial communities, OTUs with a relative abundance ≥ 0.5 were stratified by gestational age. In Fig. 5B, the heatmap highlights a small subset of taxa that achieved a high relative abundance in AF samples, indicating that most detected microorganisms were low in biomass and sporadic in occurrence. This variability in microbial presence suggests that these microorganisms may be involved in specific physiological processes, potentially influencing the overall dynamics of the microbial community in AF. Among these, *C. acnes* and *S. epidermidis* stand out as the most prominent taxa, not only due to their widespread presence across all gestational periods but also because they reach high relative abundance values (> 0.75) in 22 samples, particularly between weeks 14–17 and 22–26.

Other taxa, such as *B. licheniformis*, *Lactobacillus mulieris*, and *F. periodonticum*, were detected less consistently, each appearing only in a single sample. However, in those cases, they nearly dominated the microbial community, with relative abundance values approaching 1.0, suggesting an episodic but pronounced microbial presence in specific individuals.

### Microbial composition and diversity identified by shotgun metagenomic sequencing of amniotic fluid

To complement the taxonomic insights from the16S rRNA gene sequencing, we performed shotgun metagenomic sequencing on five AF samples selected based on the presence or absence of culturable bacteria. This approach enabled a more comprehensive characterization of microbial communities, offering strain-level resolution. The sequencing results revealed a taxonomically diverse, yet low-biomass microbial community, with notable variability in microbial content across samples.

Shotgun metagenomic sequencing yielded approximately 1.2 to 8.3 million raw reads per AF sample, encompassing both microbial and host reads. A high percentage of these sequences were classified across all samples, (ranging from 76.5% to 98%; Table 3). The proportion of microbial reads varied notably, from less than 5% in AF_9 to > 60% in AF_29. Bacterial reads constituted most of the microbial content in each sample, with AF_29 and AF_35 showing particularly high percentages (59.33% and 35.19%, respectively).

**Table 3:**
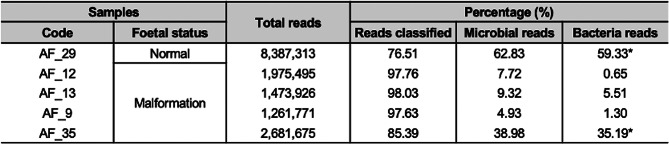
Summary of sequencing classification results for five AF samples analysed by shotgun metagenomics

The table displays the total number of reads, the percentage of classified reads, and the proportions of microbial and bacterial reads. Asterisks (*) indicate samples with the highest relative abundance in each microbial category. AF, amniotic fluid

Among the detected taxa, *Phyllobacterium* was the predominant genus in two samples, from which high-quality metagenome-assembled genomes (MAGs) were recovered. These MAGs were over 90% complete with low contamination.

**Fig. 6:**
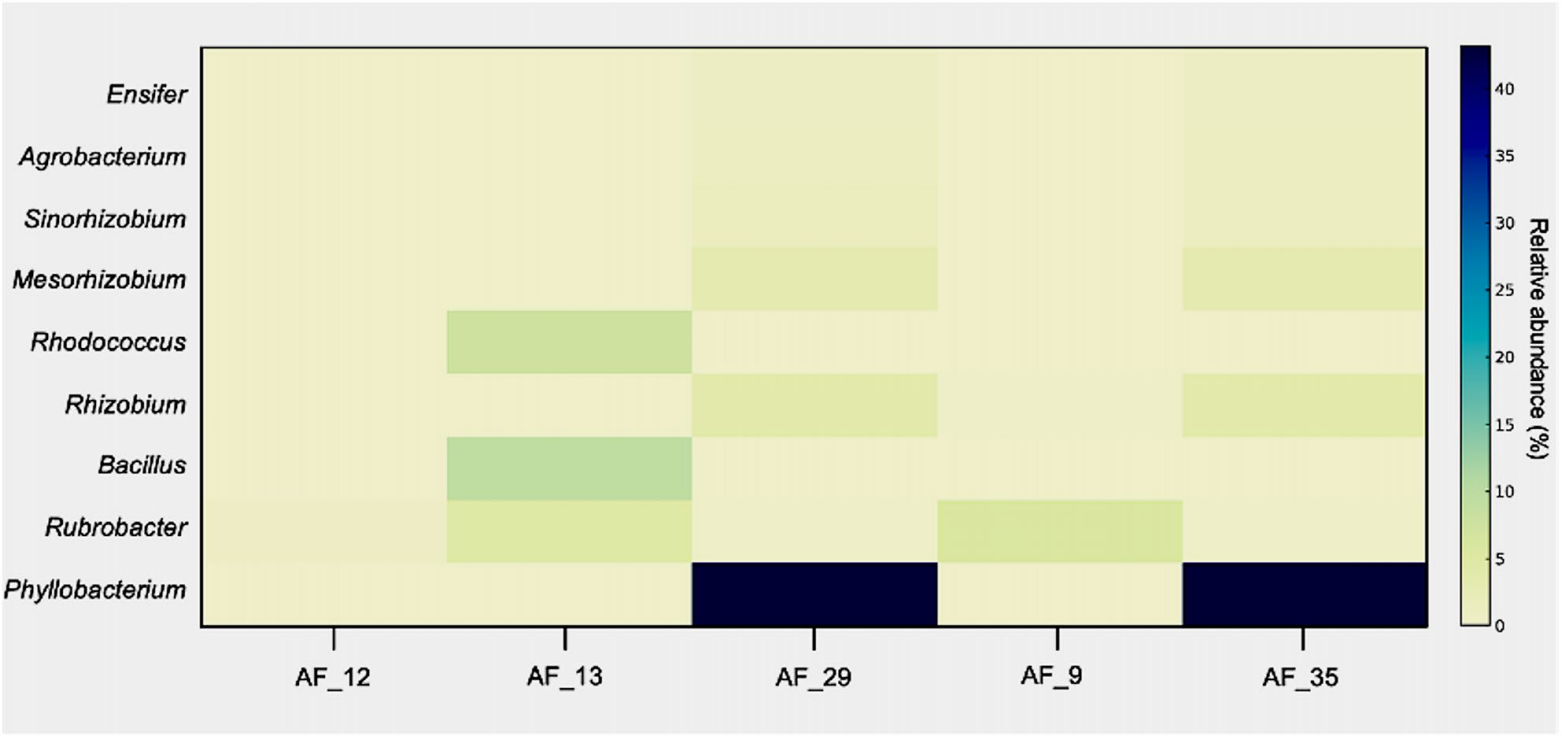
Relative abundance of dominant bacterial genera detected by shotgun metagenomics across AF samples from amniocentesis. Only genera with > 0.5% relative abundance are shown. Color intensity reflects the proportion of taxonomically classified reads assigned to each genus based on shotgun sequencing data. AF, amniotic fluid

The particular abundance of *Phyllobacterium* sp. T1293, *Phyllobacterium* sp. 628, and *Phyllobacterium zundukense* was a notable finding, suggesting they represent a significant and likely under-detected component of the microbial composition of AF. Other bacterial genera were identified at lower abundance, indicating that the microbial community was dominated by a few key species (Fig. 6).

### Impact of different clinical and procedural aspects on the presence of microorganisms in amniotic fluid

**Fig. 7:**
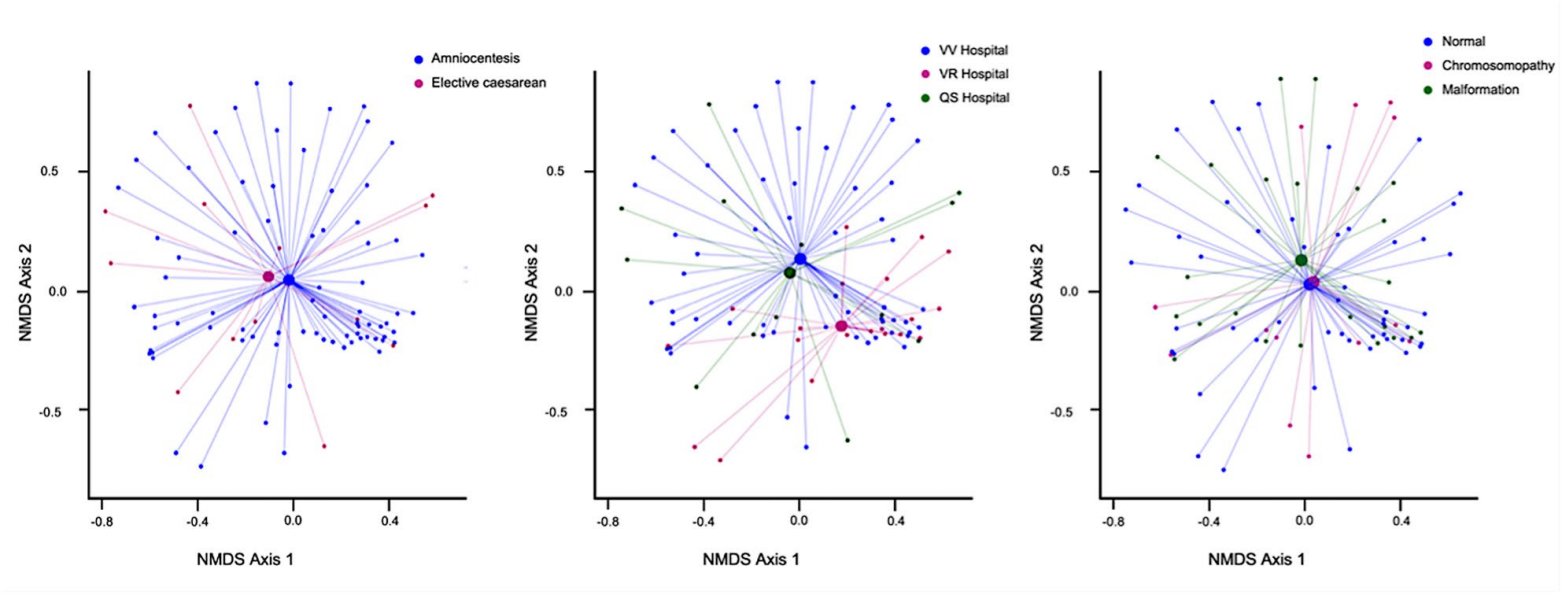
Beta diversity analysis by NMDS and PERMANOVA. Samples were grouped according to **a** gestational period (R^2^ = 0.01421; *p* = 0.1939), **b** sample collection center (R^2^ = 0.01275; *p* = 0.2796), and **c** foetal status features: normal, malformations, and chromosomopathies (R^2^ = 0.0095; *p* = 0.6262). Clustering patterns represent differences in microbial community composition among groups. NMDS, non-metric multidimensional scaling. *p < 0.05. QS, Quirón Salud; VR, Virgen del Rocío; VV, Virgen de Valme

To further investigate the overall microbial community structure across different clinical and procedural variables, we performed a beta diversity analysis using non-metric multidimensional scaling (NMDS) of the 16S rRNA gene data. As shown in Fig. 7, the ordination plots illustrate sample clustering according to (A) gestational period (amniocentesis *versus* elective caesarean sections) (R^2^ = 0.01421; *p* = 0.1039), (B) sample collection centre (R^2^ = 0.01275; *p* = 0.2796), and (C) foetal condition (normal, chromosomopathy, or structural malformation) (R^2^ = 0.0095; *p* = 0.6262). Although some visual grouping can be observed in the NMDS plots, pairwise Permutational Multivariate Analysis of Variance (PERMANOVA) analyses did not reveal statistically significant differences between any of the groups (*p* < 0.05), indicating that the global microbial composition shows substantial inter-sample variability across these conditions. No significant differences in microbial composition were detected when samples were stratified by weeks of gestational age (R^2^ = 0.1831, *p* = 0.8937), assisted *versus* spontaneous conception (R^2^ = 0.02229, *p* = 0.0767) or pregnancy outcome (R^2^ = 0.00996, *p* = 0.8202).

The analysis of culturable bacteria in AF samples revealed a significant association between isolated bacteria and the type of reproductive treatment (*n* = 10, assisted conception; *n* = 90, spontaneous conception; *p* = 0.028). However, culture positivity was not significantly associated with gestational age (*p* = 0.9017), maternal age (*p* = 0.703), foetal status (*p* = 0.1692), or pregnancy outcome (*p* = 0.7607). These findings indicate that, within this cohort, the presence of culturable bacteria was not dependent on most maternal or foetal clinical characteristics.

### Microbial diversity and intra-individual variability in diamniotic twin amniotic fluid

To evaluate the microbial composition of diamniotic AF samples, we identified bacterial species in paired amniotic sacs from three individuals using culture-based methods and 16S rRNA gene sequencing. In the case of AV_3 amniocentesis, 16S rRNA gene sequencing identified five bacterial taxa in each sac, with shared species including *B. altitudinis*, *C. acnes*, and *Escherichia coli*. Notably, culture methods detected *Solibacillus isronensis* exclusively in sac 2, whereas sac 1 yielded no culturable bacteria. These findings suggest a similar microbial community at the sequencing level, but a limited overlap in culture results between sacs. In the case of AV_60 amniocentesis, *S. epidermidis* was consistently detected by both culture and sequencing in sac 1. *C. acnes* was also detected in both sacs by at least one detection method. Sac 2 displayed greater microbial diversity via 16S rRNA gene methods, identifying six species compared with two in sac 1, suggesting a partial overlap alongside distinct differences in microbial content between the sacs. In the EC_5 elective caesarean section case, *S. epidermidis* was the sole species recovered by culture-based method (sac 1) and was also detected by 16S rRNA gene sequencing in both sacs. However, sequencing identified a broader range of taxa in sac 1 (*Haemophilus haemolyticus*, *Rothia mucilaginosa* and *Streptococcus parasalivarius*), whereas sac 2 showed fewer taxa and no culture-positive results.

### Characterization of antimicrobial peptides in amniotic fluid: association with gestational stages and microbial load

The identification of culturable microorganisms in AF samples provides a crucial context for analysing AMP levels, enabling a link between microbial load and potential health outcomes. To further investigate this relationship, we quantified the levels of HBD 1–3, HNPs 1–3 and LL-37 in randomly selected AF samples belonging to either healthy foetuses or those with abnormalities. Of these, 7 were obtained via amniocentesis and 4 via elective caesarean sections. Among the amniocentesis samples, 3 tested positive (AF_87, AF_52 and AF_53) for culturable microorganisms, while 4 (AF_83, AF_84, AF_39 and AF_68) were negative. In the elective caesarean section group, microbial cultures were positive in half of the samples (AF_124 and AF_129) and negative (AF_133 and AF_140) in the other half, illustrating the variability in microbial presence across different sampling methods.

Interestingly, HBD 1–3, and HNP 1–3 levels did not exhibit significant differences across trimesters (*p* = 0.581, *p* = 0.667, *p* = 0.412, *p* = 0.376, respectively). However, LL-37 concentrations were significantly higher in the amniocentesis samples compared to those from elective caesarean sections (*p* = 0.042), which may reflect the differing microbiological environments associated with the timing and type of delivery rather than the sample collection method itself. Notably, LL-37 consistently emerged as the predominant AMP detected in the AF samples (Table S2), indicating its potential role as a key antimicrobial agent in the intra-amniotic environment.

Regarding the associations between AMP levels and microbial composition in AF, most peptides did not exhibit statistically significant differences between samples with and without cultivable microorganisms. However, a significant difference was observed for HBD-1 (*p* = 0.018), with lower concentrations in samples testing positive for culturable bacteria. This association highlights the potential interplay between AMP and microbial communities in AF. Moreover, when grouping samples by total relative microbial abundance (threshold: >0.05%), HBD-1 concentrations were significantly lower in samples with higher microbial loads (*p* = 0.032), indicating a negative correlation that warrants further investigation. No statistically significant differences were observed for the remaining peptides under this comparison.

Stratification of the samples based on the presence of specific genera revealed additional significant findings (Table S2). Samples positive for *Staphylococcus* in culture showed significantly reduced concentrations of HBD-1 (*p* < 0.001), HBD-3 (*p* = 0.024), and LL-37 (*p* = 0.049), suggesting that certain bacterial genera may actively influence AMP levels. No significant associations were found between peptide levels and the presence of other cultured genera such as *Bacillus*,* Cutibacterium*,* Micrococcus*,* Paenibacillus*, or *Brevundimonas*, as well as between AMP levels and bacterial species detected through 16S rRNA gene sequencing.

## Discussion

The microbial composition of AF remains a subject of intense scientific inquiry, particularly in relation to the long-standing debate over sterility *versus* non-sterility [11, 12, 15, 17, 18, 22–28]. Emerging evidence increasingly challenges the traditional paradigm of a sterile womb, suggesting instead the presence of a diverse and dynamic microbial landscape during pregnancy. Advances in NGS increasingly support the presence of a low-biomass microbial environment in AF, even in the absence of clinical infection [18, 25, 73]. Thus, the focus has shifted from the question of sterility to understanding how these microorganisms interact within the prenatal environment. This study contributes to this understanding by highlighting the potential microbial balance in the prenatal environment and its relationship with AMP, suggesting the existence of a potential prenatal microbial regulatory axis that could influence foetal health.

In analysing the microbial profiles, we optimised culture methodologies alongside 16S rRNA gene and shotgun metagenomic sequencing to achieve a comprehensive characterization of microbial profiles in AF samples collected at various gestational stages. Our approach overcomes limitations of previous studies [12, 14, 74] by analysing microbial colonization in rigorously collected AF samples from two independent cohorts of women at different hospitals. Importantly, this study accounts for intrinsic differences in foetal status and employs stringent contamination control measures. Secondly, we explored the presence of AMP in AF, offering new potential insights into their regulatory functions in shaping the prenatal microbial landscape.

Under the indicated conditions, culturable microorganisms were detected in approximately one-third of all AF samples reinforcing the notion that the intrauterine environment is not entirely sterile. Notably, the proportion of positive cultures was significantly higher in samples from elective caesarean Sect. (55.0%) compared to those obtained via amniocentesis (29.5%). These findings are consistent with previous reports suggesting that AF may harbour microorganisms even in uncomplicated pregnancies [18, 31] and highlight a potential increase in microbial load or viability as gestation progresses towards term. Our methodological optimization was critical for enhancing the viability and successful cultivation of fastidious or low-abundance microorganisms, which are often undetectable under standard microbiological conditions. This approach minimised bacterial lysis, and the release of intracellular contents, thereby ensuring successful subsequent bacterial growth on culture plates and providing a more accurate representation of the microbial culturable AF. The consistent presence of *B. subtilis*, *C. acnes*, *M. luteus*, and *S. epidermidis* across both second trimester and term samples further supports the existence of a persistent, albeit low-biomass, microbial community throughout pregnancy. Notably, a greater number of colonies were isolated from caesarean section-derived samples under anaerobic incubation, suggesting a higher prevalence or viability of facultative anaerobes in these cases. These findings underscore the critical importance of optimised culturing techniques for heterogeneous AF samples. The microbial profiles observed in our study support the hypothesis that fluctuating oxygen levels influence the early microbial landscape, even prior to birth. The presence of both aerobic and anaerobic microorganisms in AF samples suggests that the intra-amniotic environment may exhibit dynamic or microaerophilic oxygen conditions, thereby facilitating the establishment of metabolically diverse microbial communities *in utero.* Although direct experimental evidence regarding foetal gut oxygenation remains limited, it has been proposed that the foetal gut represents a mildly oxygenated (aerobic) niche that transit to anaerobic conditions following birth [75–77]. Therefore, the bacteria present in the AF could colonize the foetal gut through the foetus´s swallowing of the AF.

We also detected the species *A. pretoriensis* and *C. funkei* in AF from uncomplicated pregnancies. Notably, the genus *Amycolatopsis* has been found in perinatal contexts in animals. Specifically, *A. pretoriensis* was previously identified in inflamed equine placentas [78], and other species from the same genus have been associated with placentitis in animals [79]. Furthermore, *C. funkei* has been identified in placental and neonatal fecal samples [80], and it has also been detected in other low-biomass microbiome studies [81]. Our detection of these taxa in these samples highlights the need for further studies to clarify their potential role in the prenatal environment.

The AF microbial composition identified by 16S rRNA gene sequencing showed a low abundance and low diversity bacterial community. The predominant bacterial phyla included Actinobacteria, Bacteroidetes, Firmicutes, Proteobacteria, and Fusobacteria. Notably, all bacterial species identified in samples from elective caesarean sections were also present in those obtained via amniocentesis, suggesting a consistent microbial presence throughout gestation. These findings highlight the complementary value of culture-dependent and culture-independent techniques, sequencing provided a broader view of the phylogenetically rich bacterial community, while culture-based approach confirmed the viability of specific microorganisms. However, it is important to acknowledge the limitations of both methods. While sequencing detects a wider range of taxa, it cannot differentiate between live and dead bacteria or cell-free forms [82], which complicates the interpretation of functional relevance. Conversely, our culturing methods, despite being optimized, may have failed to detect bacteria present at very low concentrations [83], or those with specific nutritional and/or environmental requirements of the bacteria, may not have been provided by the culture media and conditions used. The presence of circulating cell-free bacterial DNA and peptides, potentially transferred from the mother via the umbilical cord, as previously proposed [84, 85] suggests a key microbial translocation mechanism. This hypothesis aligns with reports of microbial DNA in placental and amniotic compartments and supports a role for maternal-foetal molecular exchange in shaping the prenatal immune environment [26, 86]. The occurrence of immunologically active microbial fragments may contribute to foetal immune priming, even in the absence of live microorganisms.

The presence of typical vaginal commensals such as *Lactobacillus* alongside other taxa is not unexpected, given the anatomical proximity and the potential for maternal microbial transmission [27, 87]. However, our data suggest this may be an intermittent or non-predominat occurrence, *Lactobacillus* was detected in only 5/91 samples, with just one sample exhibiting a relative abundance greater than 0.5, reaching 0.99. Supported by stringent contamination controls, these results reinforce that the microbial landscape of the amniotic environment is highly variable and dynamic, reflecting a selective and possibly transient microbial exchange, potentially shaded by differential dynamics or selective pressures across pregnancies.

The application of 16S rRNA gene sequencing broadened the scope of microbial detection, revealing additional phyla such as Bacteroidetes and Fusobacteria that were not recovered through culture-based methods. These findings reinforce the concept that culture and sequencing offer complementary perspectives, particularly in low-biomass environments. Nonetheless, the overlap between taxa identified by culture and those detected via sequencing was limited to seven, emphasizing methodological biases and intrinsic differences in sensitivity and taxonomic resolution between both approaches.

Among the most frequently isolated taxa were *C. acnes* and *S. epidermidis*, found consistently across gestational periods. While both species are commonly regarded as skin commensals and potential contaminants, their repeated detection across multiple samples, clinical sites, and sequencing platforms combined with low read counts in negative controls strongly suggests their biological relevance rather than a simple contamination. Specifically, although both species were identified in negative controls, the mean number of reads from the negative controls was significantly lower (15.75 and 51.75 *C. acnes* and *S. epidermidis*, respectively compared to the mean total number of reads of 53,043.82 found in the analysed AF samples). Further supporting their presence as viable organisms, these species were successfully cultivated from AF samples, and notably, no growth was observed in our culture negative controls. These findings, combined with their repeated isolation, strongly indicate that they represent a genuine component of the amniotic microbial community rather than being contaminants. Their presence could, therefore, play a role in foetal immune priming or microbial programming. Future research is needed to elucidate the precise biological function of these microorganisms and validate our hypothesis of foetal immune priming.

Our investigation into the dominant microbial communities in AF revealed no clear trend of microbial abundance or dominance with advancing gestational age. This finding supports the hypothesis of a transient, low-diversity bacterial presence within the amniotic environment and indicates that microbial load in AF is highly variable between individuals, limited to a few dominant taxa per sample. Ultimately, our results suggest episodic microbial exposure rather than stable colonization, emphasizing a potential dynamic prenatal microbial landscape.

Our shotgun metagenomic sequencing analysis provided complementary taxonomic information and enabled higher resolution for specific taxa, most notably *Phyllobacterium* spp., from which high-quality MAGs were recovered. These MAGs were over 90% complete with low contamination, confirming the high quality of our genomic data. The abundance of *Phyllobacterium* sp. *T1293*, *Phyllobacterium* sp. *628*, and *Phyllobacterium zundukense* suggest that multiple members of this genus are well-adapted to the mildly oxygenated intra-amniotic environment. Metabolic reconstruction further indicates that *Phyllobacterium* exhibits a versatile metabolic profile requiring only basic inorganic salts (e.g., phosphate, sulfate, Fe²⁺, Mg²⁺, Mn²⁺, Ca²⁺), trace amounts of certain amino acids (e.g., L-serine, L-phenylalanine, L-proline), and vitamins such as thiamin. Additionally, it can metabolize a wide range of carbon sources. This low dependency on complex nutritional factors, combined with its ability to thrive under mild aerobic conditions, provides a compelling biological explanation for its persistence in this unique environment. This finding strongly suggests that its presence is not incidental, but rather a significant and likely under-detected microbial component of the AF.

Our comprehensive analysis of AF microbial communities across diverse clinical and procedural variables did not reveal conclusive insights into the nature of intrauterine microbial colonization, specifically regarding its association with these variables. Despite visual trends observed in NMDS ordination plots, beta diversity analysis did not demonstrate statistically significant differences in overall microbial composition across gestational period, sample collection centre, foetal condition, or gestational age. This finding is highly significant as it suggests that the global microbial profile within the amniotic environment in our studied cohort is largely independent of these major clinical and obstetric factors, challenging the notion that maternal or foetal characteristics directly lead to substantial shifts in the overall microbial landscape of the AF [24]. However, a significant association between bacterial isolation and the type of reproductive treatment was observed, suggesting that assisted reproductive technologies may influence the translocation or detectability of culturable bacteria in the amniotic environment.

The inclusion of AF samples from twin pregnancies constitutes a novel and significant aspect of our study, as this dual methodological approach remains largely unexplored in the existing literature in twins. Notably, we observed intra-individual microbial discordance between paired amniotic sacs in diamniotic pregnancies, suggesting localised differences in microbial exposure despite shared maternal conditions. While *C. acnes* and *S. epidermidis* were consistently detected in both sacs, unique taxa were identified in only one sac per pair, indicating a lack of microbial homogeneity even within the same intrauterine environment. Our NMDS analysis confirmed that diamniotic twins did not exhibit a more similar microbial composition than randomly selected sample pairs, in agreement with Park et al. (2023) [88]. This highlights intra-individual variability in microbial composition within diamniotic pregnancies and underscores the enhanced sensitivity of 16S rRNA gene sequencing compared to culture methods.

Our final aim was to correlate the intrinsic antibacterial activity in AF with the microbial colonization of each sample by examining AMP. Prior research shows that AMP like defensins and cathelicidins typically change concentration throughout gestation [48]. We found that LL-37 was consistently the most abundant AMP across the second and third trimesters, with higher concentrations in amniocentesis samples compared to those from elective caesarean sections. This suggests potential differences in AMP related to the gestational stage. Furthermore, we observed elevated HBD-1 levels in samples lacking culturable microorganisms, suggesting an antimicrobial function in preserving possible amniotic balance. Similarly, HBD-3 concentrations were significantly higher in samples negative for microbial growth. The significantly reduced concentrations of HBD-1, HBD-3, and LL-37 in *Staphylococcus*-positive AF samples suggest a possible interaction between this specie and the AMP host. Specifically, these results could indicate a selective suppression or degradation of these key AMP in the presence of certain bacterial taxa. Collectively, our results may support the potent antimicrobial nature of AMP, demonstrating their effectiveness in inhibiting microbial proliferation within the amniotic environment. However, further assessment in larger sample sizes is needed to confirm these results.

The limitations of this study are essentially threefold. First, the inherent low-biomass nature of the AF microbial community presents a considerable challenge for NGS analysis, being necessary stringent control to reliably distinguish authentic microbial signals from unavoidable reagent-based background contamination (the “kitome”). Second, despite our optimized approach, the culture-dependent methodology fundamentally underestimates true microbial prevalence. This is a recognized limitation in such studies, attributed to the difficulty in isolating and propagating fastidious, anaerobic, or currently unculturable organisms. Third, the absence of detailed data on maternal comorbidities can contribute to the inter-individual variability observed in the microorganisms within the AF samples, given that maternal health status can influence the host microbiome.

The key future challenge is to move from microbial detection to functional elucidation and clinical impact. This involves defining the biological function of key prenatal bacteria, validating the foetal immune priming hypothesis, and determining the long-term health impact of this prenatal microbiota and its proposed regulatory axis with AMP.

## Conclusions

Our findings indicate that viable bacteria and/or their genetic material may reach the prenatal environment before birth, challenging the notion of a sterile womb. Although the underlying regulatory mechanisms require further investigation, our results suggest their presence may be shaped by maternal and foetal immune responses, particularly via AMP. This study, by its comprehensive multi-methodological approach and unique inclusion of twin samples, provides key insights into the dynamic and complex nature of the intrauterine microbial landscape. Future research will be essential to elucidate the direct impact of this microbial interaction on foetal health outcomes.

## Supplementary Information

Below is the link to the electronic supplementary material.

Supplementary Material 1: Supplementary Figure S1-S2 and Supplementary Tables S1-S2.

## Data Availability

The datasets generated during the current study can be found in the Sequence Read Archives, under accession PRJNA1288569, https://dataview.ncbi.nlm.nih.gov/object/PRJNA1288569?reviewer=magaofe0a5tbid1opgu28qc8ba.

## Abbreviations

AF: Amniotic fluid
AMP: Antimicrobial peptides
BHI: Brain Heart Infusion
CB: Columbia Blood
HBD-1: Human Beta-Defensin 1
HBD-2: Human Beta-Defensin 2
HBD-3: Human Beta-Defensin 3
HNPs: Human Neutrophil Peptides
MA: MagAttract
MC: MacConkey
NGS: Next-Generation Sequencing
NMDS: Non-Metric Multidimensional Scaling
OTU: Operational Taxonomic Unit
PERMANOVA: Permutational Multivariate Analysis of Variance
PFP: PowerFecal Pro
