# Supplementary material for: Unveiling balanced prenatal microbial colonization in amniotic fluid through an integrated culture and sequencing approach
Source: J Transl Med. 2026 Jan 9;24:273. doi: 10.1186/s12967-025-07601-0 (PMC12918369; doi:10.1186/s12967-025-07601-0)

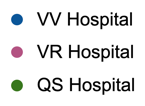
 A B C D


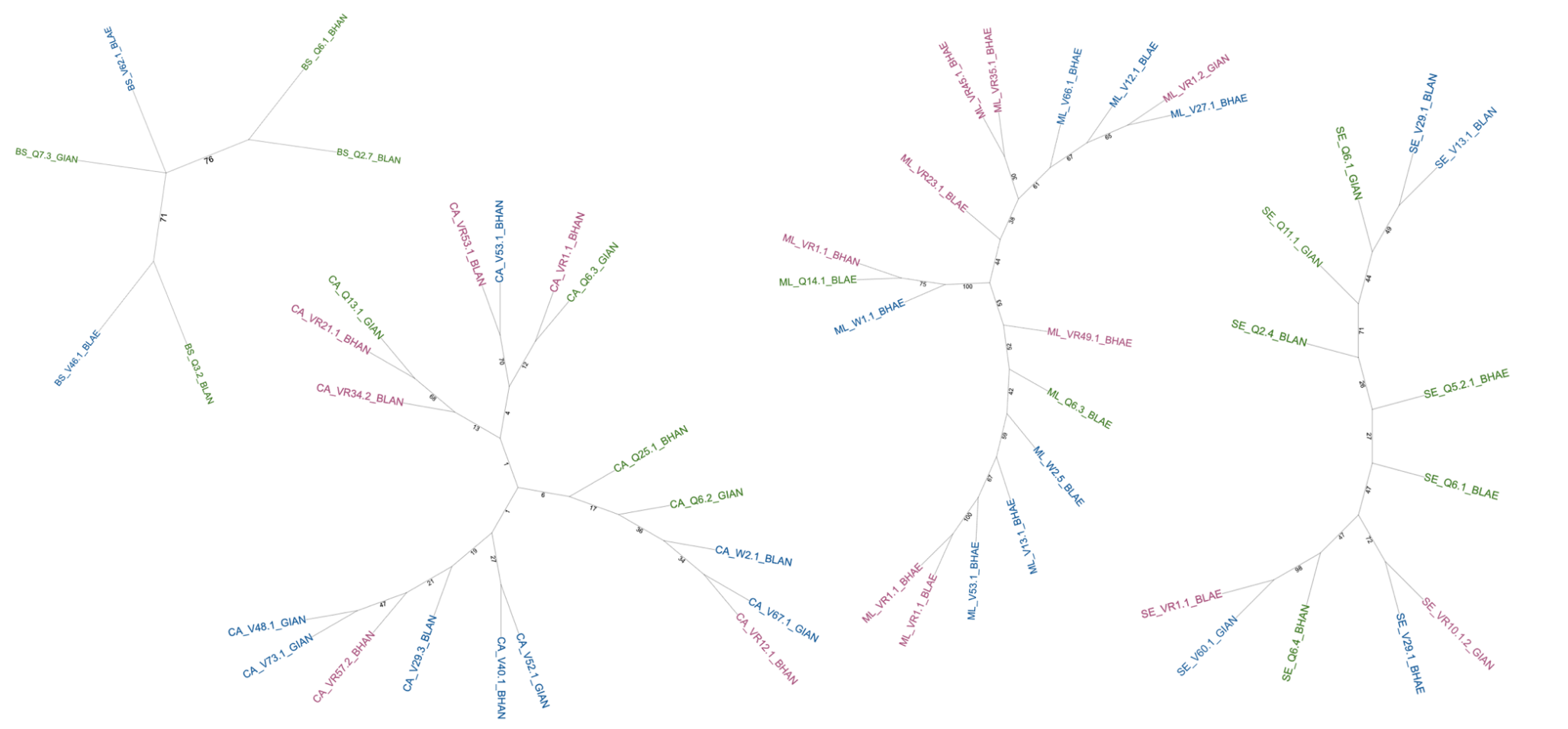


**Supplementary Fig. S1** Phylogenetic trees of four common isolated bacterial species from AF samples. The phylogenetic trees were constructed using the Neighbor-Joining method based on 16S rRNA gene sequences. **a** *Bacillus subtilis;* **b** *Cutibacterium acnes;* **c** *Micrococcus luteus;* **d** *Staphylococcus epidermidis*. Individual isolates are labeled and color-coded by collection center. Branch values indicate bootstrap support (1,000 replicates). AF, amniotic fluid; QS, Quirón Salud; VR, Virgen del Rocío; VV, Virgen de Valme.

**
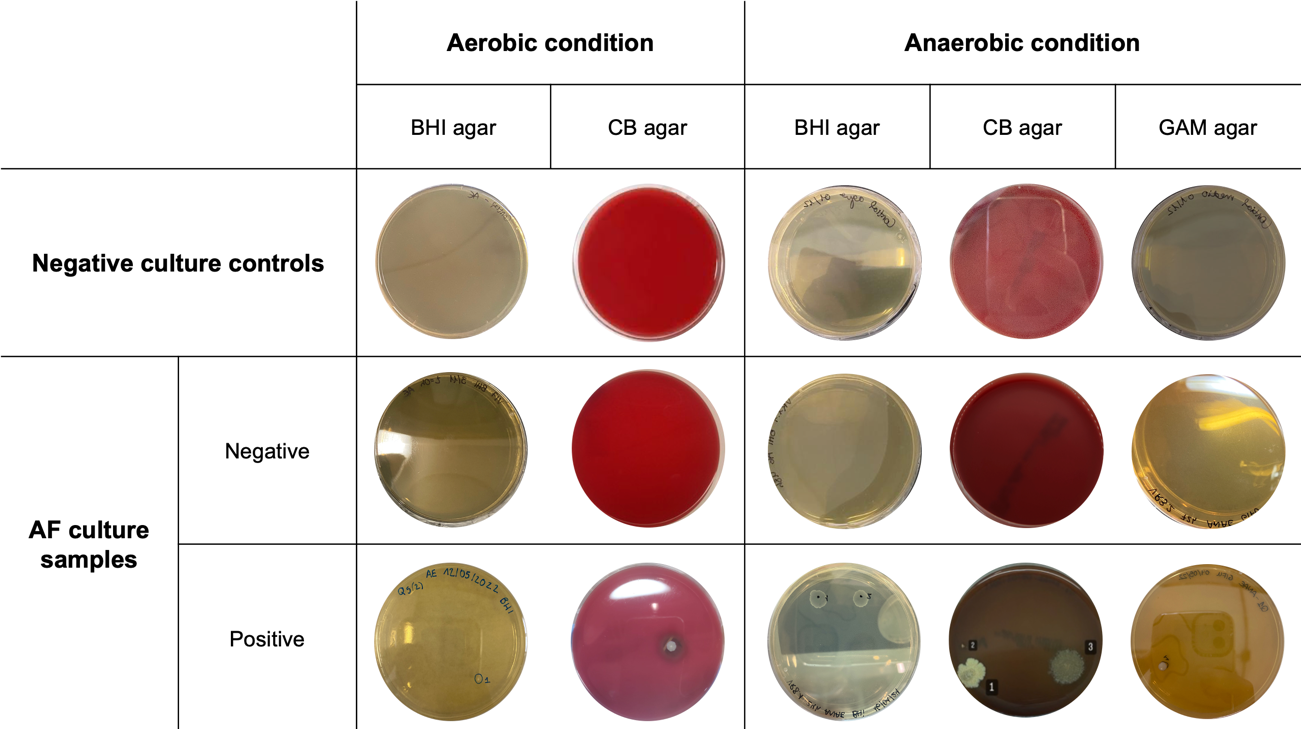
**

**Supplementary Fig. S2** Culture plates images from AF samples and negative controls grown under aerobic and anaerobic conditions. The upper row shows negative culture controls, confirming the absence of bacterial contamination. From left to right, the middle row displays AF samples (AF_18, AF_23, AF_89, AF_126, AF_83) with no bacterial growth, while the bottom row shows AF samples (AF_128, AF_14, AF_51, AF_29, AF_123) with positive bacterial growth. BHI, CB and GAM agar culture media were used to detect microorganisms. AF, amniotic fluid; BHI, Brain Heart Infusion; CB, Columbia Blood; GAM, Gifu Anaerobic Medium.

**Supplementary Table S1** Clinical characteristics of pregnant women undergoing amniocentesis. **a** Pregnancies with normal foetuses. **b** Pregnancies with foetuses diagnosed with chromosomopathies. **c** Pregnancies with foetuses presenting malformations. Most samples corresponded to deliveries at a gestational age of over 37 weeks’, except for AV_46 (34 weeks’), AV_73 and AV_56 (35 weeks’), and AV_32 and AW_6 (36 weeks’). AC, assisted conception; NA, not available; SC, spontaneous conception; SD, standard deviation; VD, vaginal delivery; w, week; y, year.

a

| **Foetal samples** | | **Participant** | **Pregnancy** | | | |
| --- | --- | --- | --- | --- | --- | --- |
| **Code** | **Gestational age, w**  *mean ± SD (20.6 ± 4.7)* | **Age, y**  *mean ± SD (33.9 ±5.9)* | **Single/Twin** | | **AC/SC** | **Interruption**  **of pregnancy** |
| AV_15 | 19 | 39 | Twin | Monoamniotic | SC | No |
| AW_3 | 22 | 28 |  |  |  |  |
| AR_27 | 22 | 33 |  |  |  |  |
| AV_31 | 18 | 36 |  | Diamniotic |  | Yes |
| AV_24 | 16 | 43 | Single | | AC | No |
| AV_10 | 17 | 32 |  |  |  |  |
| AV_39 | 19 | 36 |  |  |  |  |
| AR_31 | 27 | 46 |  |  |  |  |
| AV_29 | 16 | 39 |  |  | SC |  |
| AV_61 | 18 | 33 |  |  |  |  |
| AV_26 | 16 | 37 |  |  |  |  |
| AW_5 | 17 | 40 |  |  |  |  |
| AV_18 | 19 | 38 |  |  |  |  |
| AV_14 | 16 | 40 |  |  |  |  |
| AV_17 | 16 | 36 |  |  |  |  |
| AV_42 | 16 | 22 |  |  |  |  |
| AV_50 | 16 | 25 |  |  |  |  |
| AV_75 | 16 | 37 |  |  |  |  |
| AV_43 | 17 | 31 |  |  |  |  |
| AV_58 | 17 | 35 |  |  |  |  |
| AV_23 | 18 | 38 |  |  |  |  |
| AV_62 | 18 | 30 |  |  |  |  |
| AV_68 | 18 | 36 |  |  |  |  |
| AV_6 | 19 | 18 |  |  |  |  |
| AV_69 | 19 | 37 |  |  |  |  |
| AW_7 | 20 | 36 |  |  |  |  |
| AV_63 | 20 | 35 |  |  |  |  |
| AV_5 | 21 | 28 |  |  |  |  |
| AV_16 | 21 | 20 |  |  |  |  |
| AV_45 | 21 | 36 |  |  |  |  |
| AV_57 | 21 | 22 |  |  |  |  |
| AV_73 | 21 | 40 |  |  |  |  |
| AV_52 | 22 | 28 |  |  |  |  |
| AV_55 | 22 | 31 |  |  |  |  |
| AV_56 | 22 | 33 |  |  |  |  |
| AV_65 | 27 | 35 |  |  |  |  |
| AV_72 | 28 | 36 |  |  |  |  |
| AV_71 | 33 | 34 |  |  |  |  |
| AR_3 | 15 | 38 |  |  |  |  |
| AR_11 | 17 | 25 |  |  |  |  |
| AR_1 | 22 | 37 |  |  |  |  |
| AR_25 | 22 | 46 |  |  |  |  |
| AR_8 | 26 | 30 |  |  |  |  |
| AR_20 | 30 | 29 |  |  |  |  |
| AR_26 | 33 | 36 |  |  |  |  |
| AR_52 | 15 | 39 |  |  |  |  |
| AR_43 | 19 | 33 |  |  |  |  |
| AR_12 | 21 | 36 |  |  |  |  |
| AV_47 | 22 | 35 |  |  |  |  |
| AR_45 | 27 | 31 |  |  |  |  |
| AR_44 | 32 | 38 |  |  |  |  |

b

| **Foetal samples** | | **Participant** | **Pregnancy** | | | |
| --- | --- | --- | --- | --- | --- | --- |
| **Code** | **Gestational age, w**  *mean ± SD (19.4 ± 3.2)* | **Age, y**  *mean ± SD (36.0 ± 4.4)* | **Single/Twin** | | **AC/SC** | **Interruption of pregnancy** |
| AV_38 | 18 | 36 | Single | | SC | No |
| AV_19 | 19 | 32 |  |  |  |  |
| AV_74 | 20 | 30 |  |  |  |  |
| AR_18 | 13 | 36 |  |  |  | Yes |
| AV_13 | 16 | 44 |  |  |  |  |
| AR_10 | 16 | 36 |  |  |  |  |
| AV_44 | 18 | 29 |  |  |  |  |
| AV_4 | 19 | 39 |  |  |  |  |
| AV_54 | 20 | 36 |  |  |  |  |
| AR_2 | 20 | 37 |  |  |  |  |
| AV_7 | 22 | 37 |  |  |  |  |
| AV_1 | 24 | 28 |  |  |  |  |
| AR_29 | 24 | 43 |  |  |  |  |
| AR_47 | 24 | 39 |  |  |  |  |
| AV_53 | 18 | 34 |  |  | AC |  |
| AV_3 | 16 | 39 | Twin | Diamniotic |  |  |
| AV_60 | 22 | 37 |  |  |  |  |

c

| **Foetal samples** | | **Participant** | **Pregnancy** | | | |
| --- | --- | --- | --- | --- | --- | --- |
| **Code** | **Gestational age, w**  *mean ± SD (21.6 ± 3.8)* | **Age, y**  *mean ± SD (33.4 ± 5.1)* | **Single/Twin** | | **AC/SC** | **Interruption of pregnancy** |
| AV_32 | 20 | 30 | Twin | Monoamniotic | SC | No |
| AR_57 | 22 | 47 |  | Diamniotic |  |  |
| AV_9 | 21 | 33 | Single | | AC | Yes |
| AV_36 | 19 | 39 |  |  | SC | No |
| AV_46 | 19 | 34 |  |  |  |  |
| AV_20 | 20 | 31 |  |  |  |  |
| AV_12 | 21 | 30 |  |  |  |  |
| AW_4 | 21 | 36 |  |  |  |  |
| AW_1 | 22 | 20 |  |  |  |  |
| AW_6 | 22 | 39 |  |  |  |  |
| AV_67 | 20 | 30 |  |  |  |  |
| AV_70 | 20 | 38 |  |  |  |  |
| AV_49 | 16 | 30 |  |  |  |  |
| AV_34 | 18 | 32 |  |  |  |  |
| AV_40 | 18 | 30 |  |  |  |  |
| AV_27 | 20 | 28 |  |  |  |  |
| AV_28 | 20 | 32 |  |  |  |  |
| AW_2 | 21 | 31 |  |  |  |  |
| AV_21 | 22 | 38 |  |  |  |  |
| AV_37 | 22 | 41 |  |  |  |  |
| AV_8 | 21 | 32 |  |  |  | Yes |
| AR_53 | 21 | 31 |  |  |  |  |
| AV_64 | 21 | 39 |  |  |  |  |
| AV_66 | 18 | 33 |  |  |  |  |
| AV_41 | 22 | 32 |  |  |  |  |
| AV_48 | 22 | 27 |  |  |  |  |
| AV_2 | 19 | 37 |  |  |  |  |
| AV_51 | 20 | 24 |  |  |  |  |
| AV_22 | 21 | 33 |  |  |  |  |
| AV_11 | 22 | 28 |  |  |  |  |
| AR_19 | 15 | 36 |  |  |  | NA |
| AR_34 | 16 | 32 |  |  |  |  |
| AR_55 | 19 | 21 |  |  |  |  |
| AR_9 | 20 | 40 |  |  |  |  |
| AR_24 | 20 | 35 |  |  |  |  |
| AR_23 | 21 | 32 |  |  |  |  |
| AR_48 | 21 | 34 |  |  |  |  |
| AR_49 | 21 | 42 |  |  |  |  |
| AR_54 | 21 | 37 |  |  |  |  |
| AR_56 | 21 | 32 |  |  |  |  |
| AR_22 | 22 | 34 |  |  |  |  |
| AR_33 | 22 | 32 |  |  |  |  |
| AR_35 | 22 | 32 |  |  |  |  |
| AR_51 | 24 | 37 |  |  |  |  |
| AR_32 | 27 | 30 |  |  |  |  |
| AR_14 | 30 | 37 |  |  |  |  |
| AR_50 | 30 | 33 |  |  |  |  |
| AR_28 | 32 | 30 |  |  |  |  |
| AR_21 | 32 | 40 |  |  |  |  |
| AR_46 | 32 | 40 |  |  |  |  |

**Supplementary Table S2**  Concentration of AMP (ng/mL) in AF samples from second and third trimester. AF, amniotic fluid; AMP, antimicrobial peptides; HBD-1, human β-defensin 1; HBD-2, human β-defensin 2; HBD-3, human β-defensin 3; HNPs1–3, human neutrophil peptides 1–3; LOQ, limit of quantification; ND, not detected.


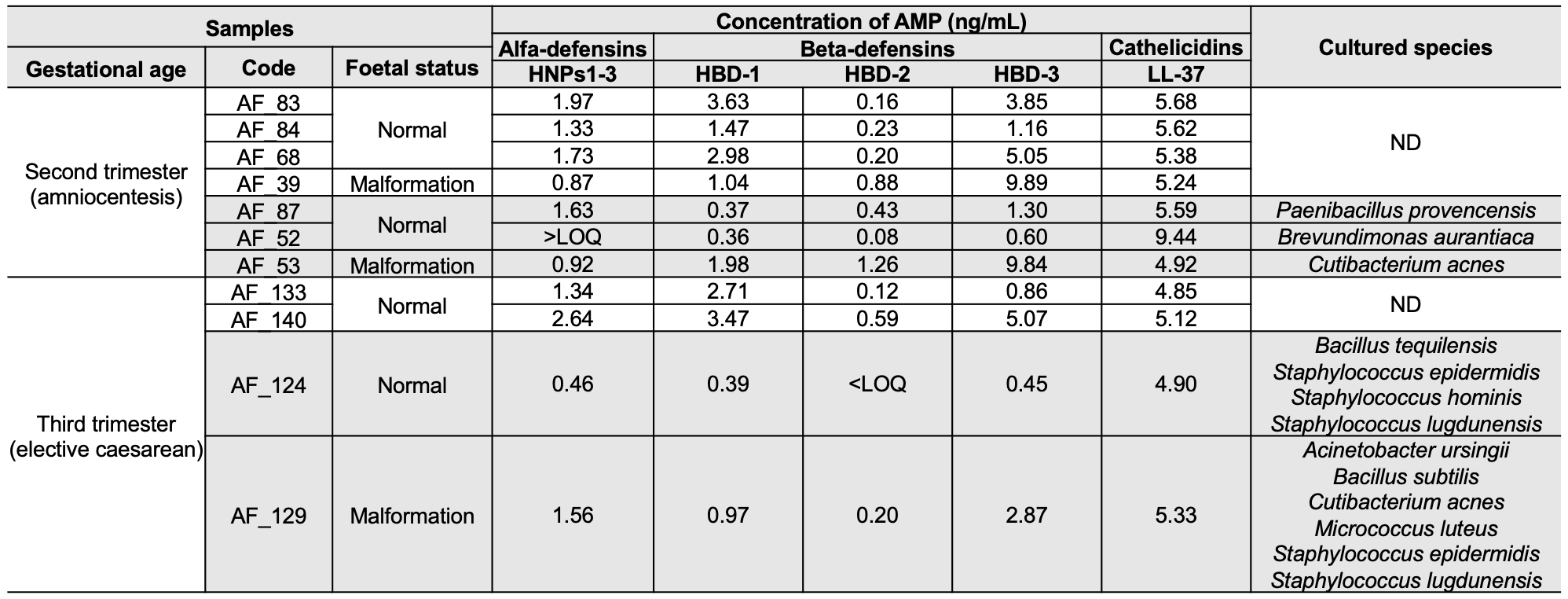

Supplement: Supplementary file 1 — Supplementary Material 1: Supplementary Figure S1-S2 and Supplementary Tables S1-S2. [file 12967_2025_7601_MOESM1_ESM.docx]
